# Hypertrophic Cardiomyopathy: Genetic Foundations, Outcomes, Interconnections, and Their Modifiers

**DOI:** 10.3390/medicina59081424

**Published:** 2023-08-04

**Authors:** Mila Glavaški, Lazar Velicki, Nataša Vučinić

**Affiliations:** 1 Faculty of Medicine, University of Novi Sad, Hajduk Veljkova 3, 21000 Novi Sad, Serbia; lazar.velicki@mf.uns.ac.rs (L.V.);; 2 Institute of Cardiovascular Diseases Vojvodina, Put Doktora Goldmana 4, 21204 Sremska Kamenica, Serbia

**Keywords:** hypertrophic cardiomyopathy, genetics, outcomes, genotype, phenotype, modifier, gene, epigenetics, comorbidities, environment

## Abstract

Hypertrophic cardiomyopathy (HCM) is the most prevalent heritable cardiomyopathy. HCM is considered to be caused by mutations in cardiac sarcomeric protein genes. Recent research suggests that the genetic foundation of HCM is much more complex than originally postulated. The clinical presentations of HCM are very variable. Some mutation carriers remain asymptomatic, while others develop severe HCM, terminal heart failure, or sudden cardiac death. Heterogeneity regarding both genetic mutations and the clinical course of HCM hinders the establishment of universal genotype–phenotype correlations. However, some trends have been identified. The presence of a mutation in some genes encoding sarcomeric proteins is associated with earlier HCM onset, more severe left ventricular hypertrophy, and worse clinical outcomes. There is a diversity in the mechanisms implicated in the pathogenesis of HCM. They may be classified into groups, but they are interrelated. The lack of known supplementary elements that control the progression of HCM indicates that molecular mechanisms that exist between genotype and clinical presentations may be crucial. Secondary molecular changes in pathways implicated in HCM pathogenesis, post-translational protein modifications, and epigenetic factors affect HCM phenotypes. Cardiac loading conditions, exercise, hypertension, diet, alcohol consumption, microbial infection, obstructive sleep apnea, obesity, and environmental factors are non-molecular aspects that change the HCM phenotype. Many mechanisms are implicated in the course of HCM. They are mostly interconnected and contribute to some extent to final outcomes.

## 1. Introduction

Hypertrophic cardiomyopathy (HCM) is the most prevalent heritable cardiomyopathy [1,2,3], with a recorded prevalence of 1 in 500 people among the general population worldwide [1,3,4,5,6,7,8,9] and recent research assessing a prevalence of up to 1 in 200 people [7,9,10,11,12,13,14]. Many people affected by HCM are undiagnosed or undertreated. Identified HCM patients form only the “tip of the HCM patients’ iceberg” [9,15]. Numerous HCM patients are still not aware of their diagnosis due to a lack of symptoms and a discreet clinical presentation, limited clinical experience with HCM, or inadequate diagnostics [15].

HCM is diagnosed by the existence of left ventricular hypertrophy (LVH) despite the lack of abnormal loading conditions causing it [16,17,18]: in adults, it is defined as left ventricular thickness of ≥15 mm [4,19]. Among first-degree family members of subjects with confirmed HCM, an unexplained left ventricular thickness of ≥13 mm is sufficient for HCM diagnosis [19]. HCM should be diagnosed as the disease secondary to mutations in the sarcomere or associated structural cardiomyocyte proteins, but only after rigorous elimination of the secondary causes of LVH and elimination of all systemic syndromes associated with LVH and mimicking HCM. Some of the secondary causes of LVH are arterial hypertension, anatomical heart disease, or medication toxicity. Other metabolic, endocrinological, neurological, neuromuscular, or autoimmune multisystem disorders and genetic syndromes can manifest characteristic HCM features [13].

Clinical manifestations and prognosis vary widely. Severe heart failure (HF) and sudden cardiac death (SCD) may arise in some HCM subjects [8,9,20,21]. SCD can rarely be the first manifestation of HCM [20]. Current therapeutic strategies (pharmacological treatment and cardiac surgical procedures) provide symptomatic treatment for HCM, leaving the causes of the disease unaddressed [19]. Treatment is usually individualized [1], and aims to alleviate symptoms and impede disease progression [19]. HCM represents a leading cause of SCD in the young [22,23,24].

The genetic basis of HCM has been traced back to 5–10 centuries ago. For this reason, HCM is not a “new” disease; however, its clinical identification is new. Contemporary HCM is to some extent a result of its often benign nature and the reproductive fitness (ability of an individual to pass on its genes to the subsequent generations) of HCM patients [15].

HCM identification and treatment have advanced notably over the previous decade—it has been converted from an odd disease with an unfavorable prognosis to a treatable disease with low mortality (<1% per year) [9,15]. With an evidence-guided approach, life expectancy has increased, leading to a notable improvement in overall quality of life. Approximately two-thirds of HCM patients now have a normal life span [13]. However, these advances are fully available only in highly developed countries and regions [15].

Research on HCM is complicated by the fact that animal models do not completely reproduce human HCM [25] due to the differences in sarcomere protein contents between them and humans [16].

### “Disease of the Sarcomere”

A sarcomere is a repeating building block and contractile unit of striated (cardiac and skeletal) muscles that coordinates muscle contraction [26,27,28,29,30]. HCM is sometimes depicted as a “disease of the sarcomere” [3,7,14], indicating that it is most often caused by mutations in genes that encode proteins associated with the cardiac sarcomere [16,31,32,33].

**Figure 1:**
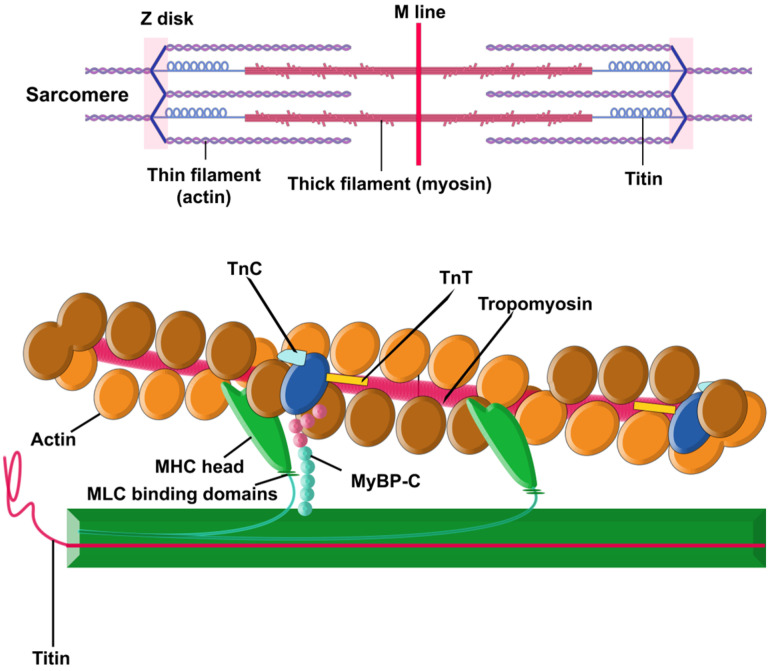
Sarcomere. Schematic illustration of a sarcomere, showcasing the essential contractile proteins, actin, and myosin, along with the structural protein titin. Additionally, the inclusion of myosin-binding protein C is deliberate, as it plays a pivotal role in finely regulating the interactions between the thin and thick filaments.

There are two principal components within the sarcomere (Figure 1): the thick filament and the thin filament. The thick filament is built by approximately 300 molecules of the motor protein myosin. Each molecule of myosin is made of two protein units of α- or β-myosin heavy chains (MHCs) and two myosin light chains (MLC). The thin filament contains repeating filamentous actin molecules, associated with the contraction-tuning troponin complex [troponin I (TnI), troponin T (TnT), and troponin C (TnC)], α-tropomyosin, and myosin-binding protein C (MyBP-C). MyBP-C links the thick and thin filaments and regulates actin–myosin interplay [29,30,31,34]. The striation pattern along a myofibril is the result of overlapping sets of actin and myosin filaments within the sarcomere [26].

Sarcomeric function is hypersensitive: disruptions in the integrity of any component (single dysfunctional sarcomeric protein, changes in structure or dynamics of sarcomere, alterations in proteolytic degradation or expression, etc.) can result in cardiomyopathies [28]. Compensatory mechanisms to overcome the defects are also sometimes activated, and some of them may, over time, become pathological too [29].

## 2. Genetic Foundation of HCM

The first HCM-associated mutation was identified in 1989 in the *MYH7* gene, which encodes MHC, a sarcomeric protein of the thick filament [7,17,31]. Thus far, >1400 HCM-associated mutations have been identified, of which approximately 90% have been found in the genes encoding proteins of the thin and thick filaments of the sarcomere (*MYH7*, *MYBPC3*, and *TNNT2*, which encode MHC, cardiac MyBP-C, and cardiac TnT) [7,12,18,35,36,37].

HCM is mainly considered to be inherited as an autosomal dominant trait [10,20,38], resulting from mutations in cardiac sarcomeric protein genes [10,20,39,40]. However, investigations revealed that the genetic foundation of HCM is much more complex than originally postulated [10,41]. The prevalence of reporting HCM has increased with the usage of advanced genetic testing and precise imaging techniques. Genetic testing allows a molecular diagnosis before ventricular hypertrophy appears [13].

Mutation carriers are usually heterozygous and carry one disease (mutant) allele and one normal allele. However, in some rare cases, mutation carriers are homozygous, and present early (childhood) onset and severe disease. HCM in heterozygous mutation carriers usually appears between the ages of 20 and 50 years. For these reasons, the first appearance of HCM seems to be predetermined by the expression of mutant proteins [7].

Approximately 60% of HCM patients have an evident familial disease (Table 1). Autosomal recessive and X-linked modes of inheritance have been described but are very uncommon. An X-linked inheritance implies the possibility that the case is actually a phenocopy condition, like Fabry disease. HCM phenocopies occur in syndromic conditions (e.g., Noonan syndrome) and in storage diseases (e.g., Anderson–Fabry disease) [16].

Pathogenic or likely pathogenic variants in sarcomeric protein genes cause 30–40% of HCM cases (depending on diagnostic criteria and approaches to variant classification) [42]. Mutations in genes *MYH7* and *MYBPC3* are the two most frequent factors, being together accountable for more than 50% of HCM cases with pathogenic variants (Table 1) [13,16,31,42,43,44]. Genetic and clinical studies have also supported the causal, but less common, pathogenic variants in other genes encoding sarcomere proteins including *TNNT2* (encoding cardiac troponin T), *TNNI3* (encoding cardiac troponin I), and *TPM1* (encoding α-tropomyosin) [17,31]. Mutations in *TNNT2*, *TNNI3*, and *TPM1* are together accountable for <10% of cases. Mutations in *ACTC1* (encoding cardiac α-actin), *MYL2* (encoding myosin regulatory light chain 2), and *MYL3* (encoding myosin essential light chain 3) are also identified as causes of HCM, though rare [16,17]. Therefore, HCM is considered to be a disease of the sarcomere; however, it can be caused by other factors as well [10,35,43].

**Table 1:** Pathogenic and likely pathogenic variants in the sarcomeric genes including novel gene variants classified according to the refined American College of Medical Genetics and Genomics (ACMG) standards and guidelines for inherited cardiac conditions (adapted from [45]).

Gene	Nucleotide Change	Amino Acid Change	ClinVar Variant Class	dbSNP	Global Frequency of Alternative Allele	Frequencies of Reference and Alternative Allele in Different Population Groups Summarized by NCBI
*TNNI3†*	c.434G>A	p.Arg145Gln	LP/P	rs397516349 [45,46]	T = 0.000019 (TOPMed)	https://tinyurl.com/272hmsky (accessed on 26 July 2023)
*MYH7†*	c.1324C>T	p.Arg442Cys	LP/P	rs148808089 [45,46]	A = 0.000023 (TOPMed)	https://tinyurl.com/5ybdf2a2 (accessed on 26 July 2023)
*MYBPC3†*	c.1484G>A	p.Arg495Gln	LP/P	rs200411226 [45,46]	T = 0.000030 (TOPMed)	https://tinyurl.com/yx44frjv (accessed on 26 July 2023)
*MYBPC3#*	c.2512G>T	p.Glu838*	ND	Novel [45,46]	ND	ND
*MYH7*	c.4130C>T	p.Thr1377Met	VUS/LP	rs397516201 [45,46]	A = 0.000004 (TOPMed)	https://tinyurl.com/3mutu3np (accessed on 26 July 2023)
*MYBPC3*	c.1000G>A	p.Glu334Lys	LP	rs573916965 [45,46]	T = 0.000098 (TOPMed)	https://tinyurl.com/bddrcys4 (accessed on 26 July 2023)
*MYBPC3#*	c.86delT	p.Phe29Serfs*10	ND	Novel [45,46]	ND	ND
*MYBPC3*	c.2459G>A	p.Arg820Gln	LP	rs2856655 [45,46]	T = 0.000015 (TOPMed)	https://tinyurl.com/2kepk5eu (accessed on 26 July 2023)
*MYBPC3†*	c.2833_2834del	p.Arg945Glyfs	P	rs397515987 [45,46]	delGC = 0.000004 (GnomAD_exome)	https://tinyurl.com/49br55fx (accessed on 26 July 2023)
*MYBPC3†*	c.1505G>A	p.Arg502Gln	P	rs397515907 [45,46]	T = 0.000019 (TOPMed)	https://tinyurl.com/4fhrbw7h (accessed on 26 July 2023)
*MYBPC3*	c.2067+1G>A	ND	ND	ND	ND	ND
*MYH7#*	c.599C>T	p.Ala200Val	ND	Novel [45,46]	ND	ND
*TNNI3*	c.485G>C	p.Arg162Pro	LP	rs397516354 [45,46]	T = 0.000011 (TOPMed)	https://tinyurl.com/4uw3zpk7 (accessed on 26 July 2023)
*MYBPC3#*	c.2272G>A	p.Gly758Ser	ND	Novel [45,46]	ND	ND
*MYBPC3#*	c.3805G>T	p.Glu1269*	ND	Novel [45,46]	ND	ND
*MYH7*	c.2608C>T	p.Arg870Cys	LP	rs138049878 [45,46]	A = 0.000024 (GnomAD_exome)	https://tinyurl.com/2n79tsm2 (accessed on 26 July 2023)
*MYH7#*	c.1350G>T	p.Lys450Asn	ND	Novel [45,46]	ND	ND
*MYBPC3*	c.2441_2443del	p.Lys814del	VUS/LP	rs727504288 [45,46]	delCTT = 0.000011 (TOPMed)	https://tinyurl.com/bdd4x5ny (accessed on 26 July 2023)
*TNNC1†*	c.23C>T	p.Ala8Val	VUS/LP/P	rs267607125 [45,46]	A = 0.000007 (GnomAD)	https://tinyurl.com/4ycbpvwu (accessed on 26 July 2023)
*MYH7#*	c.2606G>C	p.Arg869Pro	ND	Novel [45,46]	ND	ND
*TNNI3†*	c.433C>G	p.Arg145Gly	P	rs104894724 [45,46]	A = 0.000004 (TOPMed)	https://tinyurl.com/5z7fbryu (accessed on 26 July 2023)
*MYBPC3*	c.2067+1G>A	ND	ND	ND	ND	ND
*MYBPC3*	c.3490+1G>A	ND	P	rs397516020 [45,46]	A = 0.000007 (GnomAD)	https://tinyurl.com/ysefcrzu (accessed on 26 July 2023)
*MYBPC3#*	c.178G>T	p.Glu60*	ND	Novel [45,46]	ND	ND
*MYBPC3*	c.3763delG	p.Ala1255Profs*76	LP	rs786204362 [45,46]	ND	https://tinyurl.com/2pk6bpzs (accessed on 26 July 2023)
*MYH6#*	c.679dupG	p.Ala227Glyfs*24	ND	Novel [45,46]	ND	ND
*MYBPC3*	c.2067+1G>A	ND	ND	ND	ND	ND
*TNNI3†*	c.433C>G	p.Arg145Gly	P	rs104894724 [45,46]	A = 0.000004 (TOPMed)	https://tinyurl.com/5z7fbryu (accessed on 26 July 2023)
*MYL3†*	c.170C>G	p.Ala57Gly	P	rs139794067 [45,46]	T = 0.00020 (ALFA)	https://tinyurl.com/fbsuvnwh (accessed on 26 July 2023)
*MYBPC3*	c.1090+1G>A	ND	LP/P	rs727504269 [45,46]	A = 0.000004 (GnomAD_exome)	https://tinyurl.com/26fm8vmy (accessed on 26 July 2023)
*MYH6†*	c.2384G>A	p.Arg795Gln	VUS/LP/P	rs267606907 [45,46]	T = 0.000023 (TOPMed)	https://tinyurl.com/53r9um6p (accessed on 26 July 2023)
*MYBPC3*	c.2067+1G>A	ND	P	ND	ND	ND
*MYH7†*	c.1988G>A	p.Arg663His	P	rs371898076 [45,46]	T = 0.000042 (TOPMed)	https://tinyurl.com/ynuws63f (accessed on 26 July 2023)
*MYH7#*	c.1426C>T	p.Leu476Phe	ND	Novel [45,46]	ND	ND
*MYBPC3*	c.2458C>T	p.Arg820Trp	LP	rs775404728 [45,46]	A = 0.000004 (TOPMed)	https://tinyurl.com/8u3jr2zb (accessed on 26 July 2023)
*MYH7†*	c.746G>A	p.Arg249Gln	P	rs3218713 [45,46]	T = 0.000 (ALFA)	https://tinyurl.com/evrhwf8w (accessed on 26 July 2023)
*MYH7*	c.4123T>C	p.Tyr1375His	LP	rs730880790 [45,46]	ND	https://tinyurl.com/3mcajnjk (accessed on 26 July 2023)
*MYBPC3#*	c.3313_3314insGG	p.Ala1105Glyfs*85	ND	Novel [45,46]	ND	ND
*MYBPC3#*	c.3034C>T	p.Gln1012*	P	Novel [45,46]	ND	ND
*MYH7*	c.4066G>A	p.Glu1356Lys	LP	rs727503246 [45,46]	T = 0.000008 (TOPMed)	https://tinyurl.com/2uyhubyf (accessed on 26 July 2023)
*MYBPC3†*	c.2833_2834delCG	p.Arg945Glyfs*105	P	rs397515987 [45,46]	delGC = 0.000004 (GnomAD_exome)	https://tinyurl.com/49br55fx (accessed on 26 July 2023)
*MYH7†*	c.1615A>G	p.Met539Val	LP/P	rs730880930 [45,46]	ND	https://tinyurl.com/46aptmu3 (accessed on 26 July 2023)

LP: likely pathogenic; P: pathogenic; VUS: variant of uncertain significance, ND: no data, dbSNP: database of single nucleotide polymorphism, *#*: novel mutation, *†*: familial mutation; TOPMed: Trans: Omics for Precision Medicine; NCBI: National Library of Medicine; gnomAD: Genome Aggregation Database; ALFA: Allele Frequency Aggregator.

Mutations in other genes have also been described in patients with HCM (Table 2): *TTN* (titin), *ACTN1* (encoding α-actinin), *MYH6* (myosin heavy chain or α-MHC), *TCAP* (telethonin), *CSRP3* (muscle LIM protein–a Z-disc protein), *FHL1* (four-and-a-half LIM domains 1), *FLNC* (filamin C), *JPH2* (junctophilin 2), *PLN* (phospholamban), *ACTN2* (alpha-actinin-2, a Z-disc protein), *CRYAB* (crystallin alpha B), *MYOZ2* (myozenin 2, a Z-disc protein), *TNNC1* (cardiac troponin C), *TRIM55* (tripartite motif containing 55), and *TRIM63* (ubiquitin E3 ligase tripartite motif protein 63 or MuRF1) [16,31,34,42,44]. While some of these genes are likely to be causal genes for HCM, others are only associated with HCM [16].

Mutations in several other genes: *PRKAG2* (γ2-subunit of AMP kinase), *GLA* (α-galactosidase A), and *LAMP2* (lysosome-associated membrane protein 2), are detected in ~2% of cases misdiagnosed as having HCM. Mutations in these genes implicate disparate mechanisms to induce hypertrophy, but also lead to the expression of supplementary clinical phenotypes that do not appear in HCM [42]. There is a mechanistic difference between phenocopy conditions and HCM: ventricular hypertrophy in phenocopy conditions results at least partly from the storage of material (e.g., glycogen) and partly from functional defects in myocytes (e.g., impaired contraction) [16].

Causality cannot be definitely identified, especially in sporadic cases or small families. As a result of great genetic variety, the HCM-causing genes are left undefined in ~40% of cases [16].

Around 5% of HCM cases exhibit two (digenic) or more (oligogenic) causal mutations in the same or different genes [16,31,36,39]. This is not in line with the classic single gene disease definition; moreover, it suggests that a number of variants could jointly cause HCM phenotypes and each variant has a mild to moderate effect. Complex genotypes, including compound heterozygous or homozygous variants, have been described [31]. While one mutation is generally sufficient to cause HCM, it is not always the case due to variable penetrance and expression [16].

For the majority of HCM genes, both de novo and familial pathogenic variants have been described (Table 1) [31].

The percentage of novel variant detection is 35–40%, with 56% of variants found in a single family and therefore considered “private” variants [60]. Haplotype analyses of an identical variant in unrelated patients demonstrated that such genetic variants emerge independently [31]. However, many unrelated *MYBPC3* variant carriers possess the same haplotype architecture in some homogeneous subpopulations in the Netherlands [61], Finland [62], Iceland [63], Japan [64], and India [65], which demonstrate clear founder effects in HCM. Due to late-onset and benign presentations, the presence of HCM-associated founding mutations in these populations demonstrates neutral or mild negative selection [31].

In general, genetic variants range from clinically negligible to causal. Genetic variants with very large effects are considered to be accountable for autosomal dominant single-gene disorders. They have been detected using robust genetic methods, such as linkage and co-segregation analyses. Illustrations related to HCM involve genetic variants in the genes *MYH7* and *MYBPC3*. Genetic variants with clinically small or indistinguishable effects modestly impact the phenotype, but still do not cause monogenic disorders. In the middle of the spectrum lies a subset of genetic variants exerting intermediary to large effect sizes and exhibiting incomplete penetrance—their effects are affected by other non-genetic and genetic factors. Such variants cause HCM with incomplete or low penetrance [16]. Incomplete penetrance suggests that the majority of HCM mutation carriers have a low risk of pathological HCM phenotype development over their lifetime [66].

A tendency involving the higher allele frequency of *MYBPC3* and *TNNI3* variants was identified that could be a result of the reduced expressivity or penetrance of such variants (a milder disease progression compared with other sarcomeric disease gene variants). HCM cases with mutations in *MYH7* and *TNNT2* are generally younger at diagnosis (34 years) than HCM patients with mutations in *MYBPC3*, *TNNI3*, or those without mutations [67].

The frequency of particular mutations in an HCM population is markedly low: the only two exceptions are the p.Arg502Trp mutation in *MYBPC3* (identified in ~1.5–3% of HCM cases) and the p.Val762Asp mutation in *MYBPC3* (occurring in 3.9% of the Japanese population) [16]—“p.” stands for a label used in the standard nomenclature of mutations based on the amino acid sequence of the protein [68]. Other mutations appear at a frequency of <1% in the HCM population, and around half of them are detected in a single family or proband [16]. Some other variants reported to be relatively common and “hot spots” are: Arg403Gln, Arg453Cys, and Arg663His in *MYH7*; Arg92Gln, Arg92Trp, and Arg104Val in *TNNT2*; and Arg495Gln and c.1928-2A>G in *MYBPC3* [3]—the prefix “c.” stands for a label indicating the use of standard mutation nomenclature based on reference coding DNA sequences [68]. Early studies on large pedigrees with severe clinical presentations identified several “high-risk” mutations (e.g., MYH7-R403Q, MYH7-R453C25, and TNNT2-R92Q/W). However, studies on greater populations of unrelated individuals partially validated the devastating outcomes of those mutations, and were not able to find genotype–phenotype correlations for most variants [69]. Therefore, significant “hot spots” for mutations in any of the known genes do not exist [16].

An interesting aspect of sarcomeric mutations is pleiotropy—mutations in the very same gene could manifest as either HCM, dilated cardiomyopathy (DCM), restrictive cardiomyopathy, or left ventricular non-compaction cardiomyopathy. Different mutations in genes like *MYH7* and *TNNT2* lead to varied phenotypes: HCM and DCM. A possible explanation for this might be the different locations of the mutations, which would affect various protein domains, causing different inter-reactions of mutant proteins with sarcomere components and the subsequent activation of various intermediary molecular events. Similarly, causal mutations might affect the Ca^2+^ sensitivity of ATPase activity and force generation (HCM-causing mutations in thin filament proteins generally increase the Ca^2+^ sensitivity of ATPase activity and myofibrillar force generation, whereas DCM-causing mutations decrease these) [16].

In line with the variety of HCM-associated mutations, initial defects in HCM are also diverse. Initial changes in HCM include altered translation efficiency and transcription rates and alterations in the affected protein structure, as well as in the functioning of the sarcomere [16].

### 2.1. Missense and Nonsense Mutations, Allelic Imbalance, and Haploinsufficiency in HCM

In general, missense mutations alter structure and function by altering the amino acid constitution of the encoded protein [16,34]. Missense mutations usually cause the mutant protein to be integrated into the sarcomere; however, its interactions with the regular proteins hamper normal sarcomeric functioning (poison polypeptide hypothesis) [34]. Missense mutations causing structural alterations in the encoded protein might decrease the effectiveness of the sarcomere formation [16].

Allelic imbalance occurs when a higher protein expression is produced from one allele compared to another (deviating from the anticipated 1:1 expression proportion). Allelic imbalance is displayed by 25% of human genes. Protein allelic imbalance occurs if the mutation causes the production of altered protein that interferes with intrinsic protein folding, protein–protein interactions, or protein quality control, usually eventually decreasing mutant protein stability. In the case of HCM, allelic imbalance has been reported for several missense mutations in *MYBPC3*, *MYH7*, *TNNT2*, and *MYL2*. The ratio of wild-type to mutant protein differs regarding particular mutations (some specimens showed lower and some a higher proportion of mutants to the wild-type protein) [70]. Allelic imbalance is partly amended by the wild-type allele, but this allelic compensation is often incomplete. As a result, the mutation might reduce expression and cause a moderate insufficiency of the corresponding protein. In HCM, there is a myocyte-to-myocyte variation in the expression of transcripts of mutant alleles. This heterogeneity may elucidate the varying phenotypic expression of HCM [16].

As a result of insertion/deletion mutations that induce frame shift changes, altered proteins are produced. They are commonly targeted for degradation, leading to haploinsufficiency [16,34]. Haploinsufficiency represents disturbances in protein homeostasis [70]. Haploinsufficiency arises when a heterozygous mutation results in one allele of a gene being deleted or inactivated, and when a single functional copy of a gene is insufficient to maintain normal levels of protein [43,70]. The level of the functional protein does not have to be less than half of the normal, but it must be below the threshold required for proper functioning. This might also happen when a mutant protein is present but still non-functional (as in the case of in-frame mutations) [70]. Transcription and translation regulatory mechanisms prevent the biosynthesis of the truncated proteins [16].

The most pathogenic and likely pathogenic sarcomeric gene variants causing HCM encode missense residues, leading to nonsynonymous amino acid substitution with a proposed dominant negative effect. The notable exception is *MYBPC3*, wherein nonsense mutations are most often caused by frameshifts, insertions/deletions, or splice-site variants. These create a premature stop codon and hence truncated protein is produced, which further leads to the haploinsufficiency of the protein [16,31,34,42,71,72]. There exist over 300 pathogenic HCM-associated mutations in β-cardiac myosin [72].

### 2.2. Genetic Testing

Genetic testing is valuable in clarifying both HCM and phenocopy conditions diagnosis. HCM prevalence has become higher with the usage of advanced genetic testing granting a molecular diagnosis ahead of clinical diagnosis. Genetic testing is used in individuals with HCM symptoms and signs to confirm HCM diagnosis, in patients who already have a HCM diagnosis to guide genetic cascade screening in family members, and in first-degree family members estimated to be at risk of developing HCM after pre-test counseling [13]. The positive result of genetic testing supports the diagnosis of HCM in a proband, but a negative result does not exclude it [13,16].

The detection of causative mutations in a proband with HCM enables pre-symptomatic diagnosis and the clinical surveillance of relatives, as well as appropriate genetic counseling [3]. If a proband with a positive genetic test is discovered, testing for the existence of the variant in relatives (cascade screening) needs to be executed [16,17]. Mutation-positive family members should undergo regular echocardiography and electrocardiography. Mutation-negative family members can be discharged from follow-up. However, the long-term follow-up of family members is required since late-onset cardiac expression is common in HCM [73,74].

Relatives who are not carriers of the causal mutation are highly unlikely to develop HCM. The relatives of patients identified as HCM mutation carriers should be clinically examined. Phenotype-positive relatives might be diagnosed with HCM. Mutation-positive and phenotype-negative relatives should be clinically evaluated annually, or more often, if symptoms occur [16].

Many mutation-positive and phenotype-negative relatives will ultimately express the HCM phenotype. Nevertheless, because of incomplete penetrance, some mutation carriers never develop the HCM phenotype. Nonetheless, they need to be aware that they can pass the mutated gene to their offspring [16].

Genetic testing allows for the differentiation of HCM and the phenocopy conditions [16]. Genes like *TTR*, *PRKAG2*, *LAMP2*, *GLA*, and *GAA* are associated with metabolic disorders that resemble HCM, but their clinical profiles, inheritance patterns, and treatments differ from one another and from HCM [75] regarding different modes of inheritance (Fabry and Danon disease are X-linked), distinct progression (accelerated systolic dysfunction in Danon disease), distinct complications (conduction defects in *PRKAG2* disease), and specific therapeutics (enzyme replacement treatment in Fabry or Pompe disease) [73,74]. Genetic testing for HCM detects phenocopy conditions in ~3% of individuals [16].

Contemporary genetic testing for HCM fails to detect 50–60% of HCM patients [16]. Only 30% of individuals with a clinical diagnosis for HCM carry sarcomere gene mutations [74]. Up to 40% of HCM cases are isolated and sporadic, wherein the proband does not have any known HCM mutations or a family history of HCM [66]. The detection of pathogenic mutations is greater in individuals with a positive family history and in younger individuals, where it can reach 50–60%. In other individuals, it is ~30–40% [10].

Many non-sarcomeric genes linked with HCM have been increasingly added in panels for HCM, despite limited evidence of a causal role in HCM. The choice of genes included in routinely used diagnostic panels eventually lies on the spectrum between the extremes of adding only the validated genes on one side and the screening of all genes linked with HCM (irrespective of the strength of evidence) on the other [75].

The genetic screening of healthy individuals or patients with moderate hypertrophy is not advised. It can be deemed reasonable only in exceptional borderline cases [3].

## 3. Outcomes of HCM

The clinical presentations of HCM are very variable. Some mutation carriers stay asymptomatic, while others develop severe HCM, terminal HF, or SCD [7,13,76].

### 3.1. Primary Effects of Mutations in Sarcomeric-Protein-Encoding Genes

HCM mutations induce increased myofilament Ca^2+^ sensitivity. Increased tension caused by the HCM mutation initiates hypertrophic signaling, thereby causing a rise in cardiomyocyte width and cardiac mass specific for concentric hypertrophy. The degree of myofilament Ca^2+^ sensitization is mutation-specific [7].

It is suggested that HCM mutations lead to energy depletion by increasing adenosine triphosphate (ATP) turnover during the crossbridge cycle. In HCM mutation carriers, reduced myocardial efficiency already exists at an early stage of HCM before the development of hypertrophy [7].

The Ca^2+^-sensitizing effects of HCM mutations are potential substrates for ventricular arrhythmias. The HCM-mutation-induced sarcomere alterations induce adverse remodeling and cause electrophysiological perturbations. The cardiomyocytes of HCM patients show higher diastolic Ca^2+^ concentrations and the increased occurrence of cellular arrhythmias, which is explained by disease-related phosphorylation changes in Ca^2+^-handling proteins and sarcomeres [7].

### 3.2. Pathophysiologic Features of HCM

The pathophysiological features of HCM are: cardiomyocyte hypertrophy [77,78,79,80] and disarray [78,79,80,81], myocardial remodeling [79,82,83] and fibrosis [1,24,79,84,85,86], coronary microvascular dysfunction [20,79,80,87,88], myocardial ischemia [20,79,88,89,90] and hypercontractility [8,79,91,92,93], impaired myocardial relaxation [8,79,84,94], myocardial stiffness [79,81,84], and diastolic dysfunction [79,81,95,96].

Cardiac hypertrophy is a compensatory mechanism for managing biomechanical stresses and for the maintenance of proper cardiac homeostasis and output [97,98,99,100]. Several regulatory mechanisms are involved in cardiac hypertrophy, including epigenetic modifications [97,98,99]. It is not clear if cardiomyocyte hypertrophy is the cause for HCM or the result of the adaptive reactions of the heart to HCM (e.g., genetic disorders) [101].

Myocardial remodeling refers to alterations in heart architecture as a result of a variety of causes [102,103]. It comprises metabolic, morphological, or electrical alteration [104]. Cardiac remodeling refers to the rearrangement of normal cardiac structures. It represents a chronic maladaptive progressive process characterized by apoptosis, fibrosis, necrosis, matrix components remodeling, myocardial hypertrophy, ventricular dilatation, and vascular dysfunction. Multiple pathogeneses are implicated in cardiac remodeling, including genetic mutations [104].

HCM patients present coronary microvascular dysfunction (a decrease in coronary vasodilator reserve) in non-hypertrophied and hypertrophied left ventricle (LV) sections in the absence of epicardial coronary artery stenosis [20,105]. Reduction in coronary flow reserve is a good predictor of future cardiovascular events. In cases of HCM, severe impairment of microvascular function is more frequent in individuals with sarcomeric mutations [105].

HCM is associated with hypercontractility [7,96]. Impaired diastolic function with maintained or enhanced systolic function are the earliest presentations of HCM and are caused by genetic mutations [7,72]. These are apparent even before hypertrophy is detected [72]. At the myofilament level, HCM-associated hypercontractility is frequently associated with increased Ca^2+^ sensitivity of the contractile apparatus and accelerated cross-bridge cycling (cross-bridges are hypercontractile, irrespective of Ca^2+^) [106,107]. Since hypercontractility occurs before hypertrophy, it is suggested that hypercontractility at the molecular scale is a direct cause of HCM [108]. Increases in Ca^2+^ sensitivity cause hypercontractility, which further promotes hypertrophy [107].

Myocardial relaxation and myocardial stiffness have an essential role in diastolic LV function [109]. The chief regulator of intracellular stiffness in cardiomyocytes is titin. Titin plays the main role in the myofibrillar passive tension reaction to stretch in striated muscle cells. Mutations in *TTN* (the gene encoding titin) or its post-translational modifications triggered by disease result in a change in passive myocardial stiffness [110]. The proportion of the titin isoforms, N2BA:N2B, also influences diastolic dysfunction [111].

Some level of LV diastolic dysfunction is observed in almost all HCM patients [16,35,112,113]. In the context of HCM, titin plays a critical role in diastolic dysfunction in HCM: post-translational modifications of titin affect the elasticity of cardiomyocytes and diastolic properties of the LV [114]. Diastolic dysfunction is also characterized by high myocardial activation at low diastolic Ca^2+^ concentrations. High basal myofilament activation is usually sufficient to delay ventricular relaxation onset and to restrict proper ventricular filling. This high basal myofilament activation is either a direct result of changes caused by an HCM mutation, or stems from secondary alterations caused by HCM mutations. HCM-mutation-induced changes might involve: increased doses of mutant protein, increased incorporation of mutant protein into sarcomeres, isoforms switching to fetal isoforms, or post-translational modifications [113].

### 3.3. Clinical Presentations of HCM

The clinical presentations of HCM are strikingly variable [1,3,6,115,116], which sometimes makes HCM diagnosis challenging [1]. Some patients are completely asymptomatic [1,2,3,80,116] and can be identified incidentally [1], while others manifest LV outflow tract obstruction (LVOTO) [18,79,90,93,115,116], atrial fibrillation (AF) [1,9,79,115], SCD [1,2,6,79,116,117], or HF [1,3,5,6,79,118]. Most HCM cases remain asymptomatic or mildly symptomatic throughout their life [73], whereas others experience chest pain, fatigue, (exertional) dyspnea, palpitations, presyncope, and syncope [1,8,20,44,73,88,119,120,121], with dyspnea being the most common and syncope the least common [119]. The frequency of dyspnea and palpitations increases with age [73]. Chest pain may be associated with meals, exertion, or dehydration [122].

In HCM, the thickening of the left heart myocardium can vary, affecting either specific regions or involving the entire left ventricle. When the basal septum experiences significant hypertrophy, it increases the likelihood of developing LVOTO and mitral valve abnormalities.

The hypertrophied myocardium can obstruct the flow of blood from the left ventricle to the aorta, leading to LVOTO. This obstruction typically occurs at the level of the subaortic region, where the hypertrophied ventricular septum narrows the outflow tract. The narrowed passage restricts the blood flow, causing increased resistance and pressure within the left ventricle during systole. As a result, the heart needs to generate higher pressures to overcome the obstruction and pump blood effectively.

Furthermore, the hypertrophy can also affect the function of the mitral valve. In some cases of HCM, the hypertrophied myocardium can directly interfere with the normal closing and opening of the mitral valve leaflets. This can lead to abnormalities such as mitral valve systolic anterior motion (SAM) or mitral regurgitation. SAM occurs when the mitral valve leaflets are pulled towards the hypertrophied septum during systole, obstructing the left ventricular outflow and potentially causing further LVOTO.

Rhythm disorders in HCM usually consist of supraventricular and ventricular ectopic beats, and in rare cases, non-sustained or sustained ventricular tachycardia. Arrhythmias in HCM are results of mutation effects (e.g., altered calcium handling) or secondary processes, such as increased cardiomyocyte automaticity caused by hypertrophy, or re-entry caused by myocardial fibrosis. Arrhythmogenicity in HCM is also influenced by various pathophysiological factors: myocardial ischemia, altered hemodynamics, and maladaptive autonomic responses [13].

AF is the most frequent sustained arrhythmia in HCM and the general population, with rates being 4–6-fold greater in HCM than in the general population [9,123,124,125]. In HCM cases, the annual AF incidence is 2–3%, whereas lifetime prevalence is ~20–30%. AF is paroxysmal in 2/3rds of HCM patients and permanent or persistent in the remaining 1/3rd [123,124,125]. AF in HCM is caused by several factors: the genetic component, anatomical component, electrophysiological and hemodynamic abnormalities, and abnormal calcium handling, hypertrophy, as well as coronary microvascular dysfunction causing atrial ischemia [16,123,124,125,126]. However, pathophysiological and anatomical changes in HCM and AF are intertwined. AF is both the cause and effect of morphological and physiological changes in HCM [16,123,124,127].

SCD can be the first presentation of HCM [38,121,128]. HCM is the main cause of SCD in patients <35 years of age, but the precise incidence of SCD in this age is not clear [129]. The risk of SCD in individuals with HCM is <1% per year [31,38,129]. A potential indicator of an increased risk of SCD in HCM is disease-causing gene mutation [1,22,31,38,130,131,132].

## 4. Interconnections of Genetic Basis and Outcomes in HCM

HCM is a heterogeneous disease regarding both genetic mutations and the clinical course [3,10,74,118,133]. A high variety of involved mutations, the relative rareness of each of the individual mutations (private mutations are very common in families) [10,70], as well as incomplete penetrance [13,69,74,134] hamper the establishment of universal genotype–phenotype correlations. However, some trends have been identified [70].

The lack of clear genotype–phenotype associations restricts the use of genetic information in clinical management of HCM [75]. Moreover, relatives with the same mutation often have different clinical presentations, progression, and complications (some of which may remain completely phenotype-negative) [3,13,66,70,135,136].

Childhood-onset of HCM is seen to occur more often if there is a family history of early-onset HCM [135].

Similarly, just like the diversity in HCM genotypes, there is a diversity in the HCM mechanisms implicated in the pathogenesis of HCM. HCM mechanisms might be classified into groups; still, they are intertwined. The fundamental abnormalities in HCM are genetic mutations that directly affect the structure and function of sarcomeric proteins. These mutations cause primary or proximal phenotypes, such as abnormal sarcomere architecture. In response to these proximal phenotypes, secondary or intermediary phenotypes emerge, such as altered gene expression or activation of signaling pathways. These molecular alterations then lead to tertiary effects, including histological and pathological changes, which ultimately manifest as the clinical phenotypes observed in HCM, such as the hypertrophy of heart muscle and impaired cardiac function [16].

### 4.1. Genes

The presence of a mutation in some of the genes encoding sarcomeric proteins is associated with earlier HCM onset [67,135], more severe LVH [135], and worse clinical outcomes (Table 3) [3,70].

More specifically, HCM patients with identified mutations in some of the genes encoding sarcomeric proteins have worse microvascular dysfunction compared to genotype-negative individuals [20]. HCM patients having likely pathogenic or pathogenic mutations in any of the sarcomeric genes have a two-fold increased risk of unfavorable outcomes (such as death, HF, potentially fatal ventricular arrhythmias, etc.), compared to HCM cases with no mutation in sarcomeric genes. Individuals having variants of uncertain significance possess an intermediate risk. The lifetime risk of adverse outcomes in HCM is inversely associated with age at diagnosis [69].

Thin-filament mutations are associated with an often-atypical distribution of hypertrophy, prominent diastolic dysfunction, and a higher probability of restrictive progression and SCD in the course of childhood, compared with more frequent thick-filament gene mutations [69].

#### 4.1.1. *MYH7*

Mutations in the *MYH7* are usually associated with a poorer prognosis (e.g., they progress more often to end-stage HF), compared to mutations in *MYBPC3* or absent mutation [38,67,137] and an earlier disease onset [16,38].

Some critically important regions in the β myosin heavy chain are encoded by *MYH7*, such as the converter domain, which has been linked with earlier onset and malignant arrhythmias [3].

HCM patients with mutation in *MYH7* present higher C-terminal propeptide of type I procollagen (PICP) levels (compared to carriers of mutation in *MYBPC3*), which suggests increased myocardial collagen synthesis and a profibrotic state [67].

The missense mutation Arg663His in the *MYH7* gene is associated with greater risk of AF [124,125].

#### 4.1.2. *MYBPC3*

Mutations in *MYBPC3* are associated with a more moderate HCM phenotype [67,136], lower penetrance than mutations in *MYH7* [3], and elderly onset [3,10,135]. Because of its delayed onset, the reproductive age is not influenced and founder mutations (highly conserved within isolated populations) in *MYBPC3* are more frequent [3,135]. Missense mutations in *MYBPC3* are predominant in children, whereas truncation mutations are more common in adults [3]. No consensus has been identified regarding mutations in *MYBPC3* and disease severity, progression, and phenotype. The locations of truncating mutations in *MYBPC3* are not predictive of clinical outcomes either [70].

#### 4.1.3. *TNNT2*

Mutations in *TNNT2* are associated with a moderate phenotype, yet pose a high risk of SCD [3,67,136]. More recent research has shown that some mutations in *TNNT2* follow this rule, but there are also exceptions like p.Arg278 (because of its low frequency among the general population) [3]. Hearts with mutations in *TNNT2* contain less fibrosis but more severe myocyte disarray. The increased rate of SCD in patients having mutation in *TNNT2* might be a consequence of myofilament Ca^2+^ sensitization or severe myocyte disarray [67].

#### 4.1.4. Genetic Negative HCM Patients

A group of HCM patients who have no detected mutations after family member screening and exhaustive genetic testing cannot help identify an affected family member. This group has a distinct clinical course compared to sarcomere-mutation-positive patients: they are diagnosed in older age, typically male, with more moderate LVH, and more probably already have a diagnosis of hypertension [76,134].

#### 4.1.5. Gene Dosage

Gene dosage also affects prognosis in HCM [3,138]. Among HCM patients who exhibit two (digenic) or several (oligogenic) causal mutations in the same or different genes, the severity of ventricular hypertrophy appears to be more pronounced. These patients also raise the assumption that the “absence” of causal genes can be elucidated by the digenic or oligogenic character of mutations in some HCM cases [16].

Homozygosity or compound heterozygosity is linked with early onset, severe clinical manifestations, and poor outcomes (higher incidence of SCD or HF events) but is uncommon [71,135,139]. Patients with two or more sarcomeric HCM-causing mutations have an increased risk of lethal arrhythmias and poor outcomes [69].

### 4.2. Clinical Courses

#### 4.2.1. Patterns of Left Ventricular Hypertrophy

Mutations in *MYH7* and *MYBPC3* are the most frequent in HCM, involving basal septum [16]. Mutations in *TNNT2* and complex genotypes are associated with right atrial enlargement in HCM, leading to a poor prognosis. Mutations in *TNNI3* and *MYH7* are associated with the restrictive phenotype [3,135]. Mutations in *ACTC1* cause apical hypertrophy and left ventricular non-compaction [3,135].

#### 4.2.2. Ventricular Arrhythmias and Sudden Cardiac Death

HCM patients with mutations in *MYH7*, *TNNT2*, or *MYBPC3* present ventricular arrhythmias more frequently than individuals without sarcomeric mutations. Among these, the rate is highest in the *MYH7* group [67]. The risk of SCD is small among patients carrying pathogenic/likely pathogenic variants without hypertrophy. Mutations in *TNNT2* might represent an exception [10].

## 5. Disease Modifiers

**Figure 2:**
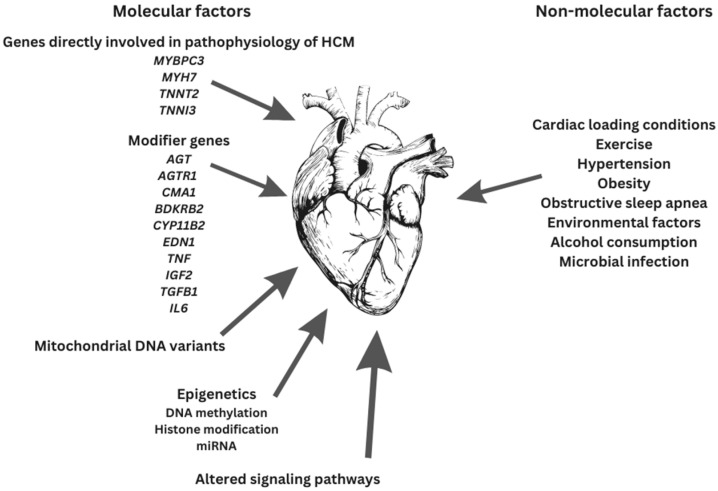
Factors contributing to final HCM phenotype.

HCM is a multifarious disease with diverse mutations, allelic imbalance, penetrance, and heart contours as well as disease modifiers, all being partly responsible for the definitive outcomes (Figure 2) [72].

Since the HCM phenotype involves the contribution and interplay of both genetic mutations and other factors (e.g., environmental factors and gene modifiers), definitive genotype–phenotype associations are still unknown [16,66,134,136]. The etiology of HCM appears to be multifactorial. Sarcomeric dysfunction might be a mandatory though not necessarily starting point in HCM pathogenesis [66].

### 5.1. Molecular Disease Modifiers

The lack of clear genotype–phenotype associations in HCM underscores the importance of discovering supplementary elements that control the progression of HCM and indicates that molecular mechanisms existing between genotype and clinical presentations may be crucial. Despite active research, molecular interactions in HCM are poorly understood [31,67,76,79].

Secondary molecular changes in pathways implicated in HCM pathogenesis and post-translational protein modifications, as well as epigenetic factors (e.g., microRNAs and small noncoding RNAs), affect histological and clinical HCM phenotypes [13,16]. HCM phenotypes are directed by several factors, each of them causing a small effect [16].

#### 5.1.1. Modifier Genes

Genetic aspects beyond causal mutations affect the phenotypes of single-gene disorders. This is especially the case with autosomal dominant diseases characterized by age-dependent onset and variable expressivity. Mutations in genes encoding contractile sarcomeric proteins cause HCM; still, the contribution of the causal mutations to the definitive phenotype could be moderate, and other genes (together with the environment) could contribute significantly [140].

The genes involved in the pathophysiology of HCM are not all the same. HCM susceptibility genes (e.g., *MYBPC3*, *MYH7*, *TNNT2*, *TNNI3,* etc.) are directly implicated in the pathophysiology of HCM, whereas modifier genes or variants are implicated in the adjustment of its phenotypic expression [140,141]. Modifier genes are neither sufficient nor necessary to cause HCM. They constitute the genetic background of individuals, and the presence of DNA polymorphisms makes a genetic background relatively individual [16,140]. The ultimate phenotype is the result of the causal mutations, environmental factors, and modifier genes [140].

Several histologic and morphologic HCM phenotypes are compensatory and regulated by numerous factors [140]. Pathogenic variants of genes involved in the regulation of cardiac hypertrophy and fibrosis may act as modifier genetic variants [16].

Gene modifier candidates for HCM are: angiotensin-1 converting enzyme-1 (*ACE*) [3,140,141], angiotensinogen (*AGT*) [3,140,141], angiotensin II receptor 1 (*AGTR1*) [3,140,141], chymase (*CMA1*) [140], bradykinin B2 receptor (*BDKRB2*) [140], aldosterone synthase (*CYP11B2*) [3,140,141], endothelin-1 (*EDN1*) [140], tumor necrosis factor α (*TNF*) [140], insulin-like growth factor 2 (*IGF2*) [140], transforming growth factor β1 (*TGFB1*) [140], interleukin 6 (*IL6*) [140], and platelet-activating factor acetylhydrolase (*PLA2G7*) [140].

Several polymorphisms in genes encoding proteins involved in the renin–angiotensin–aldosterone system (RAAS), acting alone or together, might impact the phenotypic alterations found in HCM and are considered modifiers [141]. This is because changes in “activation status” of the RAAS may lead to more prominent LVH and remodeling [31,141]. RAAS contributes to ventricular hypertrophy through circulating angiotensin effects and by local activation of RAAS in the myocardium. Polymorphisms in genes encoding proteins involved in the RAAS are associated with increased severity of LVOTO and progressive septal hypertrophy, while both of them are risk factors for adverse outcomes [141].

Angiotensin-1 converting the enzyme-1 gene (*ACE*) DD genotype is linked with the degree of LVH. However, the role of RAAS polymorphism in HCM is still debatable [3]. An insertion/deletion variant in the *ACE*, which is associated with variation in the plasma levels of ACE, modestly modifies cardiac hypertrophy and the risk of SCD in HCM [16].

#### 5.1.2. Mitochondrial DNA Variants

The heart demands a lot of energy. A single cardiomyocyte comprises hundreds of mitochondria. Mitochondrial DNA mutations have been linked with childhood HCM. Furthermore, an association between several mitochondrial haplotypes and clinical progression of cardiomyopathies has been shown. The proposed mechanisms might involve polymorphisms in mitochondrial genes, causing decreased energy efficiency. For example, haplogroup H represents a susceptibility factor whereas haplogroup J represents a protective factor for HCM. This is the result of haplogroup H having higher mitochondrial oxidative damage [this haplogroup has the highest maximal oxygen consumption (VO2max)] in contrast with haplogroup J (the lowest VO2max consumer) [3].

#### 5.1.3. Epigenetics

Modifications of gene expression in the absence of alterations in the genetic code are referred to as epigenetics [142,143]. Epigenetics refers to modifiers that have a role in switching genes “off” and “on” [142]. These changes in gene expression are mediated by DNA methylation/demethylation, histone modification, nucleosome positioning, and non-coding RNA-mediated modifications [142,143]. The effects of various environmental factors arise through alterations in the epigenome [76,143].

Along with genetic and environmental components, epigenetics is implicated in the pathophysiology of HCM. In individuals with HCM, phenotypic expression is shaped by epigenetic modifications. For instance, CpG island methylation of the cardiac troponin T gene results in genetic instability, which leads to the deamination of this region, in turn leading to mutations that later predispose for HCM. CpG sites are spots where cytosine (C) is next to guanine (G), and CpG islands are domains with numerous CpG sites [142]. There is evidence that end-stage cardiomyopathic hearts present differential methylation. Cardiac hypertrophy has also been associated with histone acetylation [3].

##### DNA Methylation

Research suggests that an increased methylation of exonic CpG sites in the *MYBPC3* can lead to deamination of methylated CpGs, which consequently contributes to mutation development. The frequentness of such mutations differs among particular genes. This mechanism of mutations can be accredited to highly methylated CpG site deamination within genes. Furthermore, frequent HCM-causing mutation due to G-to-T transversion may be induced by the binding of carcinogens like acrolein and benzo(a)pyrene diol to methylated CpG sites [142].

##### Histone Modification

Histones maintain chromatin in an active or silenced state by interreacting with DNA. Histone deacetylases 5 (HDAC5) inhibits histone deacetylases 2 (HDAC2) through deacetylation. The phosphorylation of HDAC5 occurs when the myocardium is stimulated by hypertrophic stress and the activation of HDAC2 causes myocardial hypertrophy. Thus, histone deacetylases play a critical role in cardiac hypertrophy development [142].

##### Micro RNAs (miRNAs)

miRNA is a negative regulator, operating through complementary mRNA silencing [142,143]. Concentrations of miRNA change during various phases of HCM [3,142], so there is a potential for them to serve as severity markers in the course of HCM [142]. In HCM, there are increased pro-fibrotic and pro-hypertrophic miRNAs and decreased miRNAs, leading to opposite effects. In cardiac tissue, miR-1 and miR-133 act as anti-hypertrophic factors, targeting several hypertrophic signaling molecules like transforming growth factor (TGF) β and fatty acid-binding protein (FABP). miR-29a is increased only in the plasma of patients with obstructive HCM. This is in line with a correlation between the interventricular septum size and miR-29a [142].

In this context, long non-coding RNAs (IncRNAs), which are also non-coding RNAs, play an important role. lncRNAs affect gene expression at the transcriptional and post-transcriptional levels. They are involved in the development of HCM through the regulation of chromatin remodeling and interaction with the matching miRNAs. Levels of IncRNA myocardial-infarction-associated transcript (MIAT) are inversely correlated with miR-29a expression in HCM. Patients with no fibrosis exhibit increased lncRNA-MIAT and decreased miR-29a compared to those with fibrosis. One study has suggested that IncRNA-MIAT may serve as an endogenous miRNA buffer that regulates the expression of miR-29a-3p [142].

Several microRNAs (particularly miR29a) can be used as biomarkers for interstitial fibrosis and cardiac hypertrophy [13]. There are some expectations that miRNAs may be useful as therapeutic agents for inherited cardiac diseases, as a few miRNAs appear to be heart-tissue-specific [3].

#### 5.1.4. Signal Pathways Involved in Cardiac Hypertrophy

HCM mutations directly change the structure and function of the sarcomeric proteins and biophysical characteristics of the cardiomyocyte [13]. The initial defects cause secondary (intermediary) molecular events such as the activation of Ca^2+^-sensitive, stress-responsive molecular pathways [16] and alter cellular energy balance [13]. These events produce the histological and morphological (tertiary) HCM phenotypes that are clinically presented as HCM [16]. HCM mutations can trigger other signaling pathways: mitotic and trophic factors (e.g., calcineurin), mitogen-activated protein kinases, and transforming growth factor (TGF) β pathways [13,16] and can also promote non-cardiac cells (e.g., fibroblasts). Genetic variants in the molecular pathways involved in cardiac hypertrophy and fibrosis can act as disease modifiers [13]. The intermediate molecular events are activated in other cardiac hypertrophic responses as well (e.g., pressure-overload-induced cardiac hypertrophy). Myocyte disarray, interstitial fibrosis, and cardiac hypertrophy are results of the intermediate molecules and pathway activations [16].

For instance, the development of cardiac hypertrophy involves a complex set of pathways comprising dozens of receptors, ligands, transcriptional effectors, and cytoplasmic signal amplifiers. Some of the signal transduction molecules include MAPK, TGF, tyrosine kinase, fibroblast growth factor (FGF), insulin-like growth factor (IGF), protein kinase C (PKC), and c-Jun N-terminal kinase (JNK). These pathways additionally alter gene transcription, thereby causing alterations in protein synthesis, which then leads to cardiac hypertrophy. An example of extracellular signal transduction from the plasma membrane to the nucleus is the calcineurin/nuclear factor of the activated T-cell (NFAT) pathway. Cyclic guanosine monophosphate (cGMP)-dependent protein kinase-1 (PKG-1) is a significant downstream effector of cGMP signaling in cardiomyocytes. Recent research has shown interaction between the phosphoinositide 3-kinase (PI3K) (Akt signaling pathway causing physiological cardiac hypertrophy) and the protein kinase C beta 2 (PKCβ2) (causing pathological cardiac hypertrophy) [142].

### 5.2. Non-Molecular Disease Modifiers

Cardiac loading conditions, exercise [31,141], diet [31], alcohol consumption [141], microbial infection [76], environmental factors, and other diseases are non-molecular aspects that change the HCM phenotype. This proposes that the HCM phenotype is an outcome of the complex interplay between molecular and non-molecular factors [13,31].

#### 5.2.1. Changes in Loading Conditions

Since cardiac hypertrophy is a result of the original defects caused by mutations, it is expected that changes in loading conditions (like systemic arterial hypertension) increase the severity of hypertrophy. Heavy physical activity is also expected to promote cardiac hypertrophy in cases where mutations in genes encoding sarcomere proteins are already present [16,141].

#### 5.2.2. Exercise

Regular physical activity can improve the symptoms of HCM patients by improving LV diastolic dysfunction and enhancing microcirculation via improvement in endothelial function [129].

The optimum exercise prescription in HCM remains unknown, but it appears that the benefits of regular physical activity can outweigh the risks of physical inactivity. Initiating regular exercise training during the early phases of HCM can even benefit genotype-positive relatives, providing a window of opportunity to prevent progression to the HCM phenotype through favorable physiological adaptations [144].

However, HCM is the most frequent cause of SCD in young athletes. Hence, patients should avoid engaging in intense physical activities and participating in competitive sports [137]. According to the current guidelines, the HCM diagnosis represents a cause for disqualification from competitive sports. Intensive physical activity is associated with an increase in LVH and can serve as a trigger for malignant arrhythmias [3].

#### 5.2.3. Hypertension

The hypertensive heart is generally not easy to differentiate from HCM. Furthermore, hypertension can have a negative effect on HCM progression by promoting additional LVH through diverse mechanisms, like neuroendocrine activation and increased afterload [137].

Hypertension enhances the extent of LVH via the induction of molecular pathways leading to cardiomyocyte hypertrophy, similarly to intense physical exercise [3].

Numerous HCM patients develop hypertension during their life. Women usually have a prior diagnosis of hypertension more often than men [134].

#### 5.2.4. Obesity

Obesity is linked with increased HCM penetrance, a more severe phenotype, and more rapid and worse disease progression [134,145]. Excess weight is associated with increased LV hypertrophy and mass [3,134]. LVOTO is more frequent in overweight patients—it is observed in more than 50% HCM patients with body mass index > 30 [134].

Obesity affects the myocardium and amplifies the detrimental effects of sarcomere mutations via multiple mechanisms: LV remodeling, inflammation, perfusion defects, hemodynamic changes, neurohumoral activation, and metabolic perturbations. Obesity impacts the HCM phenotypic expression and progression by affecting cardiac function independently of mutation-induced effects. Furthermore, it was also hypothesized that obesity intensifies mutation-induced pathogenic effects. In obese HCM patients, the heart appears to dilate exceedingly to increase stroke volume. This suggests that the presence of a sarcomeric mutation decreases the myocardial capacity to handle increased obesity-related demands [145].

In general, long-term obesity is closely linked to cardiac remodeling, characterized by left ventricular hypertrophy, cardiac fibrosis, and diastolic dysfunction, eventually progressing to heart failure. The disproportionate deposition of metabolically active visceral adipose tissue (VAT) and pericardial fat drives an increase in cardiac output and workload, resulting in the enlargement of the left ventricle to meet the heightened energy requirements [146].

Obese patients with HCM more often present a significant LV outflow tract obstruction and are more symptomatic, as evaluated by the New York Heart Association (NYHA) functional class. They show decreased physical activity tolerance and capacity compared to non-obese individuals. Obesity is linked with an increased LV mass index, LV cavity enlargement, greater posterior wall thickness, and larger LA diameter [145].

There is a high prevalence of obesity in HCM patients. It is shown that while HCM patients are advised to regularly carry out non-strenuous physical activity, most of them do not follow these instructions due to fear of SCD, mental discomfort, or misinterpreted medical advice. This non-compliance contributes to an increase in body weight, and the sedentary lifestyle further negatively impacts HCM progression [145].

Increased body weight predisposes individuals to develop HCM: high body mass index (BMI) in young adulthood is a predictor of HCM development later in life, and each 1-unit increase in BMI increases the risk of being diagnosed with HCM by 9% [145].

Obesity is closely related to diabetes. The clinical course is worse in diabetic patients with HCM. Compared to non-diabetic patients, diabetic HCM patients more often display LA enlargement, diastolic dysfunction, and mitral regurgitation. HCM patients with type 2 diabetes mellitus additionally show lower exercise capacity and worse NYHA functional class symptoms [145].

In obese individuals, cardiac workload and cardiac output are increased due to increased circulating blood volumes (and increased afterload, if hypertension coincides). HCM cardiomyocytes containing mutant protein compulsorily increase mitochondrial workload as a consequence of high ATP usage by inefficient sarcomeres. Moreover, missense mutations in HCM impair length-dependent activation, which probably limits the cardiac contractile reserve when augmented preload appears. Persisting obesity-promoted preload elevation can consequently decrease the threshold for compensatory hypertrophy in HCM. There is an association between the quantity of truncal fat and septal thickness. The amount of epicardial fat is associated with the N-terminal prohormone of brain natriuretic peptide levels [145]. Obesity represents an independent risk factor for HF in HCM cases [147].

Obesity may lead to cardiac adiposity, which in turn can induce endothelial dysfunction and local myocardial inflammation. The intramyocardial accumulation of fat is a source of cytokines and proinflammatory adipokines, which further contribute to vasodilation impairment, cardiac remodeling, and stiffening. Cardiac adiposity is associated with lipotoxicity, which is shown to be detrimental to cardiomyocyte homeostasis. The epicardial fat volume is associated with the extent of cardiac hypertrophy, level of circulating biomarkers indicating myocyte injury, and diastolic dysfunction severity. Clinical manifestations observed in obese HCM patients may therefore be explained by myocardial adiposity [145].

Another factor related to obesity is hypertrophic stimuli driving LV remodeling, which affects molecular mechanisms. This prevents the incorporation and accumulation of mutant proteins [145].

In obese and diabetic patients, sympathetic nervous system overactivity is often seen. A high adrenergic drive occurs in symptomatic HCM with LVOTO. Chronic overstimulation of the β-adrenergic receptor leads to its downregulation and pathway desensitization. Likewise, adrenergic receptor stimulation can add to the already high adrenergic drive in HCM, causing β-adrenergic signaling pathway impairment and additional impairment of cardiomyocyte function. β-adrenergic overstimulation also induces oxidative stress and thereby disrupts cardiomyocyte homeostasis [145].

Hyperlipidemia and hyperinsulinemia in obese and diabetic individuals cause a higher transfer of fatty acids to the myocardium. As time goes by, the heart metabolism no longer has substrate flexibility and relies more on fatty acid oxidation. Glucose oxidation is more efficient than ATP production through fatty acid oxidation regarding the amount of ATP molecules generated per oxygen atom used. In the HCM cardiomyocyte, this lack of balance in energy production can deteriorate the already existing mutation-related perturbations in the mitochondrial regeneration of ATP [145].

Also, in obese individuals, despite the increase in fatty acid oxidation, the uptake of fatty acids surpasses the capacity of fatty acid oxidation and leads to intracellular lipid deposition, some of which may be transformed into toxic lipid species causing lipotoxicity. Lipotoxicity is connected with oxidative stress, mitochondrial dysfunction, endoplasmic reticulum stress, inflammation, and apoptosis. Lipid excess increases the amount of acetyl-CoA precursors, which has been detected in failing hearts. Increased acetyl-CoA negatively regulates autophagy. Inhibited autophagy reduces mutant protein clearance, therefore increasing the mutant protein dose [145].

Hyperglycemia promotes the generation and myocardial accumulation of glycation end-products, which induces diastolic dysfunction and inflammation [145].

#### 5.2.5. Obstructive Sleep Apnea

Obstructive sleep apnea and HCM are a particularly frequent and destructive combination—obstructive sleep apnea is found in 32–71% of HCM patients [134,137]. This wide range is probably the result of varied definitions of sleep-disordered breathing. In comparison to HCM patients without obstructive sleep apnea, those with obstructive sleep apnea are older and have greater limiting symptoms, exercise capacity, and more hypertension [134]. Patients diagnosed with both HCM and obstructive sleep apnea show worse structural and functional heart impairment [137], increased prevalence of AF [134,137], and worse quality of life [137]. Obstructive sleep apnea and HCM share some key pathophysiological mechanisms: myocardial hypertrophy, LA dilation, and overstimulation of the sympathetic nervous system [137].

#### 5.2.6. Sex

Sex is suggested to be an important modifying factor in HCM [3], and could provide an explanation for some part of the clinical heterogeneity of HCM [134]. Women have a tendency to develop HCM later in life, but when it occurs, affected women are more symptomatic [3,134]. They are 6–13 years older at diagnosis than men [134].

A possible explanation for a delay in disease onset could be estrogen, which could have a role in cardiac hypertrophy inhibition through epigenetic modulations [67,134]. On the other hand, the exposure of cardiomyocytes to androgens can produce hypertrophy. Studies have shown that males usually predominate over the life course up to 60 years of age, when females become the more prevailing—pre-menopausal women might be more protected against developing HCM compared to men. However, another study has proposed that the high number of younger women (<50 years) diagnosed with HF may indicate that menopause is unlikely to have a role in HCM [134].

Women are more likely to be sarcomere-variant-positive, while men are more likely to be sarcomere-variant-negative [134]. Male patients predominate in the *MYBPC3*, *TNNT2*, and mutation-negative groups. A well-balanced sex ratio in the *MYH7* group might be the result of higher penetrance compared to the *MYBPC3* or sarcomeric mutation negative group [67].

Maximum LV wall thickness, left atrium (LA) diameter, and LV cavity size are often greater in men. However, the female heart is smaller than the male heart, and after correction for body surface area, they show relatively greater maximal LV wall thickness, LA diameter, and LV cavity size. Current HCM diagnostic criteria do not take this into account—women need to have relatively greater hypertrophy to meet at least 15 mm maximal LV wall thickness (current diagnostic criteria). Reduced heart size also explains the postponement in recognizing HCM; thus, more prominent symptoms are found in women. However, there is no difference between men and women in severe hypertrophy prevalence (LV wall thickness ≥ 30 mm) [134].

Women have worse diastolic dysfunction, a higher prevalence of an obstructive phenotype, and more prominent HF symptoms. Regardless of similar extent of hypertrophy, women more often manifest dynamic LVOTO (≥30 mmHg) than men. Women also have more mitral regurgitation, which is likely associated with reduced cavity size. Women more frequently have HF symptoms, especially fatigue, exertional dyspnea, chest pain, and palpitations, and NYHA functional classes III to IV, compared to men [134].

Women more frequently undergo alcohol septal ablation and septal myectomy, which is probably related to the higher prevalence of obstruction and HF symptoms and to the increased age at diagnosis [134].

#### 5.2.7. Environmental Factors

Comparing phenotypes in monozygotic twins, who share identical genetic sequences, reveals that while hypertrophic cardiomyopathy (HCM) shows consistent morphologic features and clinical development in identical twins, there is significant variation in left ventricular wall thickness and fibrosis amounts, suggesting the influence of environmental and epigenetic factors on HCM progression. Additionally, consistent left atrial sizes in some twin pairs suggest that defective ventricular relaxation due to sarcomeric dysfunction may directly contribute to the observed phenotype [76,148].

Another identical adult twin pair with HCM was reported as being extraordinarily similar considering their clinical course and morphological features. However, there were no differing environmental influences between the twins—they reported no notable differences in diet, exercise, or lifestyle habits and showed no evidence of exposure to radiation or chemicals [149].

## 6. Scientific Advancements

Scientific advancements have led to the development of novel strategies for studying genetic diseases, including hypertrophic cardiomyopathy (HCM). In vitro genetic disease models, specifically 3D human engineered heart tissues and CRISPR/Cas9 editing, have emerged as powerful tools for investigating the mechanisms underlying HCM [150,151]. These models provide valuable insights into disease pathogenesis, drug testing, and personalized medicine, ultimately contributing to the development of improved diagnostics and targeted therapies for patients with HCM.

In vitro genetic disease models offer a powerful platform to study the pathogenesis of HCM. These models are generated by reprogramming patient-derived cells into induced pluripotent stem cells (iPSCs), which can then be differentiated into cardiomyocytes [150,151]. By incorporating patient-specific genetic mutations, researchers can recreate the disease phenotype in a controlled laboratory environment. This approach enables the investigation of disease mechanisms, drug testing, and the evaluation of potential therapeutic interventions, providing valuable insights into HCM.

The use of 3D human engineered heart tissues in HCM research has already yielded important insights. These models can recapitulate disease-specific features, such as increased cardiomyocyte size, altered contractile properties, and impaired calcium handling. Researchers can study the effects of specific genetic mutations on sarcomeric function, cellular signaling pathways, and electrical properties of the cardiac tissue. Additionally, the incorporation of multiple cell types, including fibroblasts and endothelial cells, allows for the examination of the intricate interactions between different cardiac components and their contribution to HCM pathogenesis.

One of the significant advantages of in vitro genetic disease models is their potential for personalized medicine. By utilizing patient-specific iPSCs, researchers can tailor experimental conditions to an individual’s genetic background. This approach offers the opportunity to evaluate the efficacy of existing drugs or test novel therapeutics on a patient’s own cells, thereby advancing the development of personalized treatment strategies for HCM.

Adeno-associated viral (AAV) vectors provide safe, long-term gene transfer in a wide variety of animal organs, including the heart. The serotypes AAV6, AAV8 and AAV9 transduce cardiomyocytes preferentially following systemic administration and provide uniform gene delivery throughout the cardiac muscle. Yadav et al. conducted an in vivo study that explored AAV9-S15D-RLC gene therapy in the treatment/mitigation of the HCM phenotype in mice caused by malignant mutation [152].

Various cardiomyopathy models have been created using CRISPR [153]. In a study by Mosqueira et al., CRISPR/Cas9 was utilized to create 11 isogenic variants of an HCM-causing mutation in the *MYH7* gene. These variants were generated in three different hiPSC/hESC lines, which were then differentiated into cardiomyocytes for further analysis. The resulting cardiomyocytes displayed typical characteristics of HCM at the cellular level, including hypertrophy, excessive multi-nucleation, and sarcomeric disarray. Functional assessments revealed energy depletion, abnormalities in Ca^2+^ handling, arrhythmias, and reduced contractility. Importantly, the study demonstrated the feasibility of pharmacologically rescuing arrhythmias in these cells [151,153].

The detection of disease-causing abnormally spliced mRNA in HCM patients enhances the accuracy of genetic diagnostics but also paves the way for novel RNA-targeted therapeutic approaches. Among these approaches, splice-switching antisense oligonucleotides and short interfering RNAs show significant promise. While safety and delivery challenges must be addressed through further advancements, RNA-targeting drugs offer immense potential for treating HCM [32].

## 7. Challenges and Future Perspectives

While 3D human engineered heart tissues hold great promise, challenges remain. These include improving the maturity and complexity of the model to better mimic the human heart’s physiological conditions. Additionally, scaling up the production of these tissues and standardizing protocols are critical for widespread adoption. Future research efforts should focus on refining the model’s fidelity, integrating advanced imaging techniques for functional analysis, and establishing robust validation methods. The application of molecular phenotyping in deciphering genotype–phenotype associations, cellular pathophysiology, and early drug discovery also holds great promise for HCM. Machine learning, as an effective tool in analyzing highly complex medical data, may also bring additional insights into genotype–phenotype associations and modifiers.

Although there are some trends, there are many unknown factors influencing the development and progression of HCM as well. Each of the aforementioned relations should be investigated in more detail. For each of them, the extent of contribution to final outcomes should be assessed. Ultimately, the outcomes of the combinations of these factors should be studied in more detail.

## 8. Conclusions

Both the genetic foundation and clinical course of HCM are complex. The lack of clear genotype–phenotype associations in HCM underscores the importance of discovering supplementary elements that control the progression of HCM. Different HCM mechanisms are implicated in the pathogenesis of HCM and they are intertwined. Diverse mutations, gene dosage, and allelic imbalance as well as penetrance are all partly responsible for the definitive outcomes. The presence of mutations in *MYH7*, *MYBPC3*, and *TNNT2* affects outcomes in HCM. Molecular mechanisms might also change the course of HCM. Modifier genes or variants, mitochondrial DNA variants, epigenetics, and different signal pathways are implicated in the adjustment of phenotypic expression in HCM. Exercise, diet, alcohol consumption, microbial infection, hypertension, obstructive sleep apnea, obesity, cardiac loading conditions, and environmental factors are non-molecular aspects that change the HCM phenotype. They are mostly intertwined and contribute to some extent to the final outcomes.

## Figures and Tables

**Table 2:** Non-sarcomeric genes associated with HCM, encoded protein’s function and mode of inheritance (adapted from [47]).

Gene	Function of the Encoded Protein	Mode of Inheritance	References
*CSRP3*	Regulation of myogenesis; maintenance of myocyte cytoskeleton; mechano-signaling and transduction	AD and AR	[48,49]
*FHL1*	Biomechanical sensing; regulation of sarcomere stiffness, hypertrophy, ion channels	X-linked	[50,51]
*FLNC*	Crosslinking of actin filaments and interaction with Z-disc and sarcolemma	AD	[52,53,54]
*JPH2*	Coupling of transverse-tubule-associated L-type Ca^2+^ channels with RYR2	AD	[55]
*PLN*	Regulation of sarco/endoplasmic reticulum Ca^2+^ ATPase activity	AD and AR	[56,57]
*TRIM63*	Regulation of sarcomeric protein degradation	AD and AR	[58,59]

AD: autosomal dominant; AR: autosomal recessive.

**Table 3:** Main genes involved in HCM, their frequencies [67,95], and impact on clinical outcomes.

Mutation in Gene	Frequency	Impact on Clinical Outcomes
*MYH7*	14%	- Earlier onset - Severe HCM phenotype - Poorer prognosis (progress more often to end-stage HF or malignant arrhythmias)
*MYBPC3*	20%	- Diagnosed in older age - Moderate HCM phenotype
*TNNT2*	2%	- Moderate HCM phenotype - Pose a high risk of SCD

## Data Availability

Not applicable.

## References

[1] Geske J.B., Ommen S.R., Gersh B.J. (2018). Hypertrophic cardiomyopathy: Clinical update. JACC Heart Fail..

[2] Zegkos T., Tziomalos G., Parcharidou D., Ntelios D., Papanastasiou C.A., Karagiannidis E., Gossios T., Rouskas P., Katranas S., Paraskevaidis S. (2022). Validation of the new American College of Cardiology/American Heart Association Guidelines for the risk stratification of sudden cardiac death in a large Mediterranean cohort with Hypertrophic Cardiomyopathy. Hell. J. Cardiol..

[3] Sabater-Molina M., Pérez-Sánchez I., Hernández del Rincón J.P., Gimeno J.R. (2018). Genetics of hypertrophic cardiomyopathy: A review of current state. Clin. Genet..

[4] Firth J., Medical Masterclass contributors (2019). Cardiology: Hypertrophic cardiomyopathy. Clin. Med..

[5] Cao Y., Zhang P.Y. (2017). Review of recent advances in the management of hypertrophic cardiomyopathy. Eur. Rev. Med. Pharmacol. Sci..

[6] Antunes M.O., Scudeler T.L. (2020). Hypertrophic cardiomyopathy. Int. J. Cardiol. Heart Vasc..

[7] Van der Velden J., Stienen G.J.M. (2019). Cardiac disorders and pathophysiology of sarcomeric proteins. Physiol. Rev..

[8] Borsari W., Davis L., Meiers E., Salberg L., McDonough B. (2022). Living with hypertrophic cardiomyopathy: A patient’s perspective. Future Cardiol..

[9] Maron B.J., Desai M.Y., Nishimura R.A., Spirito P., Rakowski H., Towbin J.A., Dearani J.A., Rowin E.J., Maron M.S., Sherrid M.V. (2022). Management of hypertrophic cardiomyopathy: JACC state-of-the-art review. J. Am. Coll. Cardiol..

[10] Bonaventura J., Polakova E., Vejtasova V., Veselka J. (2021). Genetic testing in patients with hypertrophic cardiomyopathy. Int. J. Mol. Sci..

[11] Semsarian C., Ingles J., Maron M.S., Maron B.J. (2015). New perspectives on the prevalence of hypertrophic cardiomyopathy. J. Am. Coll. Cardiol..

[12] Prondzynski M., Mearini G., Carrier L. (2019). Gene therapy strategies in the treatment of hypertrophic cardiomyopathy. Pflug. Arch..

[13] Wolf C.M. (2019). Hypertrophic cardiomyopathy: Genetics and clinical perspectives. Cardiovasc. Diagn. Ther..

[14] Younger J., Lo A., McCormack L., McGaughran J., Prasad S., Atherton J.J. (2020). Hypertrophic cardiomyopathy: Challenging the status quo?. Heart Lung Circ..

[15] Maron B.J., Rowin E.J., Maron M.S. (2018). Global burden of hypertrophic cardiomyopathy. JACC Heart Fail..

[16] Marian A.J., Braunwald E. (2017). Hypertrophic cardiomyopathy: Genetics, pathogenesis, clinical manifestations, diagnosis, and therapy. Circ. Res..

[17] Jordà P., Oudit G.Y., Tadros R. (2022). Unraveling the genetic substrate and phenotypic variability of hypertrophic cardiomyopathy: A role for desmosome gene variants?. Can. J. Cardiol..

[18] Pai S.L., Chadha R.M., Logvinov I.I., Brigham T.J., Watt K.D., Li Z., Palmer W.C., Blackshear J.L., Aniskevich S. (2022). Preoperative echocardiography as a prognostic tool for liver transplant in patients with hypertrophic cardiomyopathy. Clin. Transplant..

[19] Elliott P.M., Anastasakis A., Borger M.A., Borggrefe M., Cecchi F., Charron P., Hagege A.A., Lafont A., Limongelli G., Authors/Task Force members (2014). 2014 ESC Guidelines on diagnosis and management of hypertrophic cardiomyopathy: The Task Force for the Diagnosis and Management of Hypertrophic Cardiomyopathy of the European Society of Cardiology (ESC). Eur. Heart J..

[20] Aguiar Rosa S., Rocha Lopes L., Fiarresga A., Ferreira R.C., Mota Carmo M. (2021). Coronary microvascular dysfunction in hypertrophic cardiomyopathy: Pathophysiology, assessment, and clinical impact. Microcirculation.

[21] Zampieri M., Salvi S., Fumagalli C., Argirò A., Zocchi C., Del Franco A., Iannaccone G., Giovani S., Ferrantini C., Palinkas E.D. (2023). Clinical scenarios of hypertrophic cardiomyopathy-related mortality: Relevance of age and stage of disease at presentation. Int. J. Cardiol..

[22] Hayashi T. (2020). Hypertrophic cardiomyopathy: Diverse pathophysiology revealed by genetic research, toward future therapy. Keio J. Med..

[23] Weissler-Snir A., Allan K., Cunningham K., Connelly K.A., Lee D.S., Spears D.A., Rakowski H., Dorian P. (2019). Hypertrophic cardiomyopathy–related sudden cardiac death in young people in Ontario. Circulation.

[24] O’Hara R.P., Binka E., Prakosa A., Zimmerman S.L., Cartoski M.J., Abraham M.R., Lu D.Y., Boyle P.M., Trayanova N.A. (2022). Personalized computational heart models with T1-mapped fibrotic remodeling predict sudden death risk in patients with hypertrophic cardiomyopathy. eLife.

[25] Ueda Y., Stern J.A. (2017). A one health approach to hypertrophic cardiomyopathy. Yale J. Biol. Med..

[26] Squire J. (2019). Special issue: The actin-myosin interaction in muscle: Background and overview. Int. J. Mol. Sci..

[27] Bassiouni W., Ali M.A.M., Schulz R. (2021). Multifunctional intracellular matrix metalloproteinases: Implications in disease. FEBS J..

[28] Henderson C.A., Gomez C.G., Novak S.M., Mi-Mi L., Gregorio C.C. (2017). Overview of the muscle cytoskeleton. Compr. Physiol..

[29] Cimiotti D., Budde H., Hassoun R., Jaquet K. (2021). Genetic restrictive cardiomyopathy: Causes and consequences—An integrative approach. Int. J. Mol. Sci..

[30] Martin T.G., Kirk J.A. (2020). Under construction: The dynamic assembly, maintenance, and degradation of the cardiac sarcomere. J. Mol. Cell. Cardiol..

[31] Teekakirikul P., Zhu W., Huang H.C., Fung E. (2019). Hypertrophic cardiomyopathy: An overview of genetics and management. Biomolecules.

[32] Ribeiro M., Furtado M., Martins S., Carvalho T., Carmo-Fonseca M. (2020). RNA splicing defects in hypertrophic cardiomyopathy: Implications for diagnosis and therapy. Int. J. Mol. Sci..

[33] Solomon T., Filipovska A., Hool L., Viola H. (2021). Preventative therapeutic approaches for hypertrophic cardiomyopathy. J. Physiol..

[34] Akhtar M., Elliott P. (2018). The genetics of hypertrophic cardiomyopathy. Glob. Cardiol. Sci. Pract..

[35] Wijnker P.J.M., Sequeira V., Kuster D.W.D., Velden J.V. (2019). Hypertrophic cardiomyopathy: A vicious cycle triggered by sarcomere mutations and secondary disease hits. Antioxid. Redox Signal..

[36] Cui Y., Liu C., Luo J., Liang J. (2022). Dysfunctional network and mutation genes of hypertrophic cardiomyopathy. J. Healthc. Eng..

[37] Butt A.K., Alkhatib D., Pour-Ghaz I., Isa S., Al-Taweel O., Ugonabo I., Yedlapati N., Jefferies J.L. (2023). Hypertrophic Cardiomyopathy. J. Cardiovasc. Dev. Dis..

[38] Hong Y., Su W.W., Li X. (2022). Risk factors of sudden cardiac death in hypertrophic cardiomyopathy. Curr. Opin. Cardiol..

[39] Zhang M., Sun X., Wu G., Wang D., Wang L., Zhang C., Zou Y., Wang J., Song L. (2022). Effect of cis-compound variants in *MYH7* on hypertrophic cardiomyopathy with a mild phenotype. Am. J. Cardiol..

[40] Wu G., Liu J., Ruan J., Yu S., Wang L., Zhao S., Wang S., Kang L., Wang J., Song L. (2022). Deleterious rare desmosomal variants contribute to hypertrophic cardiomyopathy and are associated with distinctive clinical features. Can. J. Cardiol..

[41] Tower-Rader A., Desai M.Y. (2017). Phenotype–genotype correlation in hypertrophic cardiomyopathy: Less signal, more noise?. Circ. Cardiovasc. Imaging.

[42] Yotti R., Seidman C.E., Seidman J.G. (2019). Advances in the genetic basis and pathogenesis of sarcomere cardiomyopathies. Annu. Rev. Genom. Hum. Genet..

[43] Arif M., Nabavizadeh P., Song T., Desai D., Singh R., Bazrafshan S., Kumar M., Wang Y., Gilbert R.J., Dhandapany P.S. (2020). Genetic, clinical, molecular, and pathogenic aspects of the South Asian–specific polymorphic MYBPC3 Δ25bp variant. Biophys. Rev..

[44] Huang H., Chen Y., Jin J., Du R., Tang K., Fan L., Xiang R. (2022). CSRP3, p.Arg122*, is responsible for hypertrophic cardiomyopathy in a Chinese family. J. Gene Med..

[45] Chung H., Kim Y., Park C.H., Kim I.S., Kim J.Y., Min P.K., Yoon Y.W., Kim T.H., Lee B.K., Hong B.K. (2021). Contribution of sarcomere gene mutations to left atrial function in patients with hypertrophic cardiomyopathy. Cardiovasc. Ultrasound.

[46] Walsh R., Buchan R., Wilk A., John S., Felkin L.E., Thomson K.L., Chiaw T.H., Loong C.C.W., Pua C.J., Raphael C. (2017). Defining the genetic architecture of hypertrophic cardiomyopathy: Re-evaluating the role of non-sarcomeric genes. Eur. Heart J..

[47] Borrelli F., Losi M.A., Canciello G., Todde G., Perillo E.F., Ordine L., Frisso G., Esposito G., Lombardi R. (2023). Sarcomeric versus non-sarcomeric HCM. Cardiogenetics.

[48] Lipari M., Wypasek E., Karpiński M., Tomkiewicz-Pajak L., Laino L., Binni F., Giannarelli D., Rubiś P., Petkow-Dimitrow P., Undas A. (2020). Identification of a variant hotspot in MYBPC3 and of a novel CSRP3 autosomal recessive alteration in a cohort of Polish patients with hypertrophic cardiomyopathy. Pol. Arch. Intern. Med..

[49] Janin A., Bessière F., Chauveau S., Chevalier P., Millat G. (2018). First identification of homozygous truncating CSRP3 variants in two unrelated cases with hypertrophic cardiomyopathy. Gene.

[50] Gallego-Delgado M., Gonzalez-Lopez E., Garcia-Guereta L., Ortega-Molina M., Gonzalez-Vioque E., Cobo-Marcos M., Alonso-Pulpon L., Garcia-Pavia P. (2015). Adverse clinical course and poor prognosis of hypertrophic cardiomyopathy due to mutations in FHL1. Int. J. Cardiol..

[51] Friedrich F.W., Wilding B.R., Reischmann S., Crocini C., Lang P., Charron P., Müller O.J., McGrath M.J., Vollert I., Hansen A. (2012). Evidence for FHL1 as a novel disease gene for isolated hypertrophic cardiomyopathy. Hum. Mol. Genet..

[52] Cui H., Wang J., Zhang C., Wu G., Zhu C., Tang B., Zou Y., Huang X., Hui R., Song L. (2018). Mutation profile of FLNC gene and its prognostic relevance in patients with hypertrophic cardiomyopathy. Mol. Genet. Genom. Med..

[53] Gómez J., Lorca R., Reguero J.R., Morís C., Martín M., Tranche S., Alonso B., Iglesias S., Alvarez V., Díaz-Molina B. (2017). Screening of the Filamin C Gene in a large cohort of hypertrophic cardiomyopathy patients. Circ. Cardiovasc. Genet..

[54] Verdonschot J.A.J., Vanhoutte E.K., Claes G.R.F., Helderman-van den Enden A.T.J.M., Hoeijmakers J.G.J., Hellebrekers D.M.E.I., de Haan A., Christiaans I., Lekanne Deprez R.H., Boen H.M. (2020). A mutation update for the FLNC gene in myopathies and cardiomyopathies. Hum. Mutat..

[55] Vanninen S.U.M., Leivo K., Seppälä E.H., Aalto-Setälä K., Pitkänen O., Suursalmi P., Annala A.P., Anttila I., Alastalo T.P., Myllykangas S. (2018). Heterozygous junctophilin-2 (JPH2) p.(Thr161Lys) is a monogenic cause for HCM with heart failure. PLoS ONE.

[56] Parisi V., Chiti C., Graziosi M., Pasquale F., Ditaranto R., Minnucci M., Biffi M., Potena L., Girolami F., Baldovini C. (2022). Phospholamban cardiomyopathy: Unveiling a distinct phenotype through heart failure stages progression. Circ. Cardiovasc. Imaging.

[57] Medin M., Hermida-Prieto M., Monserrat L., Laredo R., Rodriguez-Rey J.C., Fernandez X., Castro-Beiras A. (2007). Mutational screening of phospholamban gene in hypertrophic and idiopathic dilated cardiomyopathy and functional study of the PLN-42 C > G mutation. Eur. J. Heart Fail..

[58] Salazar-Mendiguchía J., Ochoa J.P., Palomino-Doza J., Domínguez F., Díez-López C., Akhtar M., Ramiro-León S., Clemente M.M., Pérez-Cejas A., Robledo M. (2020). Mutations in TRIM63 cause an autosomal-recessive form of hypertrophic cardiomyopathy. Heart.

[59] Chen S.N., Czernuszewicz G., Tan Y., Lombardi R., Jin J., Willerson J.T., Marian A.J. (2012). Human molecular genetic and functional studies identify TRIM63, encoding Muscle RING Finger Protein 1, as a novel gene for human hypertrophic cardiomyopathy. Circ. Res..

[60] Alfares A.A., Kelly M.A., McDermott G., Funke B.H., Lebo M.S., Baxter S.B., Shen J., McLaughlin H.M., Clark E.H., Babb L.J. (2015). Results of clinical genetic testing of 2912 probands with hypertrophic cardiomyopathy: Expanded panels offer limited additional sensitivity. Genet. Med..

[61] Alders M., Jongbloed R., Deelen W., van den Wijngaard A., Doevendans P., Ten Cate F., Regitz-Zagrosek V., Vosberg H.P., van Langen I., Wilde A. (2003). The 2373insG mutation in the *MYBPC3* gene is a founder mutation, which accounts for nearly one-fourth of the HCM cases in the Netherlands. Eur. Heart J..

[62] Jääskeläinen P., Kuusisto J., Miettinen R., Kärkkäinen P., Kärkkäinen S., Heikkinen S., Peltola P., Pihlajamäki J., Vauhkonen I., Laakso M. (2002). Mutations in the cardiac myosin-binding protein C gene are the predominant cause of familial hypertrophic cardiomyopathy in eastern Finland. J. Mol. Med..

[63] Adalsteinsdottir B., Teekakirikul P., Maron B.J., Burke M.A., Gudbjartsson D.F., Holm H., Stefansson K., DePalma S.R., Mazaika E., McDonough B. (2014). Nationwide study on hypertrophic cardiomyopathy in iceland evidence of a *MYBPC3* founder mutation. Circulation.

[64] Kubo T., Kitaoka H., Okawa M., Matsumura Y., Hitomi N., Yamasaki N., Furuno T., Takata J., Nishinaga M., Kimura A. (2005). Lifelong left ventricular remodeling of hypertrophic cardiomyopathy caused by a founder frameshift deletion mutation in the cardiac Myosin-binding protein C gene among Japanese. J. Am. Coll. Cardiol..

[65] Dhandapany P.S., Sadayappan S., Xue Y., Powell G.T., Rani D.S., Nallari P., Rai T.S., Khullar M., Soares P., Bahl A. (2009). A common *MYBPC3* (cardiac myosin binding protein C) variant associated with cardiomyopathies in South Asia. Nat. Genet..

[66] Chou C., Chin M.T. (2021). Pathogenic mechanisms of hypertrophic cardiomyopathy beyond sarcomere dysfunction. Int. J. Mol. Sci..

[67] Sedaghat-Hamedani F., Kayvanpour E., Tugrul O.F., Lai A., Amr A., Haas J., Proctor T., Ehlermann P., Jensen K., Katus H.A. (2018). Clinical outcomes associated with sarcomere mutations in hypertrophic cardiomyopathy: A meta-analysis on 7675 individuals. Clin. Res. Cardiol..

[68] Ogino S., Gulley M.L., den Dunnen J.T., Wilson R.B., Association for Molecular Pathology Training and Education Committtee (2007). Standard mutation nomenclature in molecular diagnostics: Practical and educational challenges. J. Mol. Diagn..

[69] Coppini R., Santini L., Olivotto I., Ackerman M.J., Cerbai E. (2020). Abnormalities in sodium current and calcium homoeostasis as drivers of arrhythmogenesis in hypertrophic cardiomyopathy. Cardiovasc. Res..

[70] Glazier A.A., Thompson A., Day S.M. (2019). Allelic imbalance and haploinsufficiency in *MYBPC3*-linked hypertrophic cardiomyopathy. Pflug. Arch..

[71] Carrier L. (2021). Targeting the population for gene therapy with *MYBPC3*. J. Mol. Cell. Cardiol..

[72] Spudich J.A. (2019). Three perspectives on the molecular basis of hypercontractility caused by hypertrophic cardiomyopathy mutations. Pflug. Arch..

[73] Veselka J., Anavekar N.S., Charron P. (2017). Hypertrophic obstructive cardiomyopathy. Lancet.

[74] Maron B.J., Desai M.Y., Nishimura R.A., Spirito P., Rakowski H., Towbin J.A., Dearani J.A., Rowin E.J., Maron M.S., Sherrid M.V. (2022). Diagnosis and evaluation of hypertrophic cardiomyopathy: JACC state-of-the-art review. J. Am. Coll. Cardiol..

[75] Mazzarotto F., Olivotto I., Boschi B., Girolami F., Poggesi C., Barton P.J.R., Walsh R. (2020). Contemporary insights into the genetics of hypertrophic cardiomyopathy: Toward a new era in clinical testing?. J. Am. Heart Assoc..

[76] Repetti G.G., Kim Y., Pereira A.C., Ingles J., Russell M.W., Lakdawala N.K., Ho C.Y., Day S., Semsarian C., McDonough B. (2021). Discordant clinical features of identical hypertrophic cardiomyopathy twins. Proc. Natl. Acad. Sci. USA.

[77] Farrell E.T., Grimes A.C., de Lange W.J., Armstrong A.E., Ralphe J.C. (2017). Increased postnatal cardiac hyperplasia precedes cardiomyocyte hypertrophy in a model of hypertrophic cardiomyopathy. Front. Physiol..

[78] Ramachandra C.J.A., Mai Ja K.P.M., Lin Y.H., Shim W., Boisvert W.A., Hausenloy D.J. (2019). Induced pluripotent stem cells for modelling energetic alterations in hypertrophic cardiomyopathy. Cond. Med..

[79] Glavaški M., Velicki L. (2021). Shared molecular mechanisms of hypertrophic cardiomyopathy and its clinical presentations: Automated molecular mechanisms extraction approach. Life.

[80] Zhang Z.L., Xu Y.Y., Qin Z., Lu Y.Z., Liu T.D., Zhang L., Shangguan J.H., Wang W., Tang J.N., Zhang J.Y. (2022). N-terminal pro-brain natriuretic peptide and adverse outcomes in Chinese patients with hypertrophic cardiomyopathy. Biosci. Rep..

[81] MacIver D.H., Clark A.L. (2016). Contractile dysfunction in sarcomeric hypertrophic cardiomyopathy. J. Card. Fail..

[82] Sukhacheva T.V., Chudinovskikh Y.A., Eremeeva M.V., Serov R.A., Bockeria L.A. (2016). Proliferative potential of cardiomyocytes in hypertrophic cardiomyopathy: Correlation with myocardial remodeling. Bull. Exp. Biol. Med..

[83] Fernlund E., Gyllenhammar T., Jablonowski R., Carlsson M., Larsson A., Ärnlöv J., Liuba P. (2017). Serum biomarkers of myocardial remodeling and coronary dysfunction in early stages of hypertrophic cardiomyopathy in the young. Pediatr. Cardiol..

[84] Ramachandra C.J.A., Kp M.M.J., Chua J., Hernandez-Resendiz S., Liehn E.A., Gan L.M., Michaëlsson E., Jonsson M.K.B., Ryden-Markinhuhta K., Bhat R.V. (2022). Inhibiting cardiac myeloperoxidase alleviates the relaxation defect in hypertrophic cardiomyocytes. Cardiovasc. Res..

[85] Coppini R., Ferrantini C., Mugelli A., Poggesi C., Cerbai E. (2018). Altered Ca^2+^ and Na^+^ homeostasis in human hypertrophic cardiomyopathy: Implications for arrhythmogenesis. Front. Physiol..

[86] Volpe M., Liuzzo G. (2022). VANISHing the progression of cardiac abnormalities in hypertrophic cardiomyopathy with early use of valsartan?. Eur. Heart J..

[87] Yin L., Xu H.Y., Zheng S.S., Zhu Y., Xiao J.X., Zhou W., Yu S.S., Gong L.G. (2017). 3.0 T magnetic resonance myocardial perfusion imaging for semi-quantitative evaluation of coronary microvascular dysfunction in hypertrophic cardiomyopathy. Int. J. Cardiovasc. Imaging.

[88] Bakar S.N., Hayman S., McCarty D., Thain A.P., McLellan A., Wagner C., Lavi S. (2022). Invasive assessment of microvascular resistance in hypertrophic cardiomyopathy with echocardiographic correlates. Heart Lung Circ..

[89] Raphael C.E., Cooper R., Parker K.H., Collinson J., Vassiliou V., Pennell D.J., de Silva R., Hsu L.Y., Greve A.M., Nijjer S. (2016). Mechanisms of myocardial ischemia in hypertrophic cardiomyopathy: Insights from wave intensity analysis and magnetic resonance. J. Am. Coll. Cardiol..

[90] Ariss R.W., Khan Minhas A.M., Nazir S., Patel M.M., Nesheiwat Z., Mhanna M., Kayani W.T., Moukarbel G.V., Nasir K., Jneid H. (2022). Outcomes and revascularization strategies of ST-elevation myocardial infarction in patients with hypertrophic cardiomyopathy. Curr. Probl. Cardiol..

[91] Argirò A., Zampieri M., Berteotti M., Marchi A., Tassetti L., Zocchi C., Iannone L., Bacchi B., Cappelli F., Stefàno P. (2021). Emerging medical treatment for hypertrophic cardiomyopathy. J. Clin. Med..

[92] Toepfer C.N., Wakimoto H., Garfinkel A.C., McDonough B., Liao D., Jiang J., Tai A.C., Gorham J.M., Lunde I.G., Lun M. (2019). Hypertrophic cardiomyopathy mutations in *MYBPC3* dysregulate myosin. Sci. Transl. Med..

[93] Autore C., Ferrazzi P. (2022). Patients with hypertrophic cardiomyopathy are getting older. Int. J. Cardiol..

[94] Cordts K., Seelig D., Lund N., Carrier L., Böger R.H., Avanesov M., Tahir E., Schwedhelm E., Patten M. (2019). Association of asymmetric dimethylarginine and diastolic dysfunction in patients with hypertrophic cardiomyopathy. Biomolecules.

[95] Velicki L., Jakovljevic D.G., Preveden A., Golubovic M., Bjelobrk M., Ilic A., Stojsic S., Barlocco F., Tafelmeier M., Okwose N. (2020). Genetic determinants of clinical phenotype in hypertrophic cardiomyopathy. BMC Cardiovasc. Disord..

[96] Ashkir Z., Johnson S., Lewandowski A.J., Hess A., Wicks E., Mahmod M., Myerson S., Ebbers T., Watkins H., Neubauer S. (2023). Novel insights into diminished cardiac reserve in non-obstructive hypertrophic cardiomyopathy from four-dimensional flow cardiac magnetic resonance component analysis. Eur. Heart J. Cardiovasc. Imaging.

[97] Su W., Huo Q., Wu H., Wang L., Ding X., Liang L., Zhou L., Zhao Y., Dan J., Zhang H. (2021). The function of LncRNA-H19 in cardiac hypertrophy. Cell Biosci..

[98] Liang T., Gao F., Chen J. (2021). Role of PTEN-less in cardiac injury, hypertrophy and regeneration. Cell Regen..

[99] Shah A.K., Bhullar S.K., Elimban V., Dhalla N.S. (2021). Oxidative stress as a mechanism for functional alterations in cardiac hypertrophy and heart failure. Antioxidants.

[100] Ye J., Yan S., Liu R., Weng L., Jia B., Jia S., Xiong Y., Zhou Y., Leng M., Zhao J. (2023). CMTM3 deficiency induces cardiac hypertrophy by regulating MAPK/ERK signaling. Biochem. Biophys. Res. Commun..

[101] Matsuura K. (2018). Toward the development of novel therapy for hypertrophic cardiomyopathy. Int. Heart J..

[102] Schirone L., Forte M., Palmerio S., Yee D., Nocella C., Angelini F., Pagano F., Schiavon S., Bordin A., Carrizzo A. (2017). A review of the molecular mechanisms underlying the development and progression of cardiac remodeling. Oxid. Med. Cell. Longev..

[103] Turner N.A., Blythe N.M. (2019). Cardiac fibroblast p38 MAPK: A critical regulator of myocardial remodeling. J. Cardiovasc. Dev. Dis..

[104] Zhou H., Wang B., Yang Y.X., Jia Q.J., Zhang A., Qi Z.W., Zhang J.P. (2019). Long noncoding RNAs in pathological cardiac remodeling: A review of the update literature. Biomed. Res. Int..

[105] Carbone A., D’Andrea A., Sperlongano S., Tagliamonte E., Mandoli G.E., Santoro C., Evola V., Bandera F., Morrone D., Malagoli A. (2021). Echocardiographic assessment of coronary microvascular dysfunction: Basic concepts, technical aspects, and clinical settings. Echocardiography.

[106] Mamidi R., Li J., Doh C.Y., Verma S., Stelzer J.E. (2018). Impact of the myosin modulator mavacamten on force generation and cross-bridge behavior in a murine model of hypercontractility. J. Am. Heart Assoc..

[107] Ren X., Hensley N., Brady M.B., Gao W.D. (2018). The genetic and molecular bases for hypertrophic cardiomyopathy: The role for calcium sensitization. J. Cardiothorac. Vasc. Anesth..

[108] Sarkar S.S., Trivedi D.V., Morck M.M., Adhikari A.S., Pasha S.N., Ruppel K.M., Spudich J.A. (2020). The hypertrophic cardiomyopathy mutations R403Q and R663H increase the number of myosin heads available to interact with actin. Sci. Adv..

[109] Voigt J.U. (2019). Direct stiffness measurements by echocardiography: Does the search for the holy grail come to an end?. JACC Cardiovasc. Imaging.

[110] Münch J., Abdelilah-Seyfried S. (2021). Sensing and responding of cardiomyocytes to changes of tissue stiffness in the diseased heart. Front. Cell Dev. Biol..

[111] Li N., Hang W., Shu H., Zhou N. (2022). RBM20, a therapeutic target to alleviate myocardial stiffness via titin isoforms switching in HFpEF. Front. Cardiovasc. Med..

[112] Wijnker P.J.M., van der Velden J. (2020). Mutation-specific pathology and treatment of hypertrophic cardiomyopathy in patients, mouse models and human engineered heart tissue. Biochim. Biophys. Acta Mol. Basis Dis..

[113] Sequeira V., Bertero E., Maack C. (2019). Energetic drain driving hypertrophic cardiomyopathy. FEBS Lett..

[114] Wenzl F.A., Ambrosini S., Paneni F. (2021). Molecular underpinnings of myocardial stiffness in patients with hypertrophic cardiomyopathy. Int. J. Cardiol..

[115] Tuohy C.V., Kaul S., Song H.K., Nazer B., Heitner S.B. (2020). Hypertrophic cardiomyopathy: The future of treatment. Eur. J. Heart Fail..

[116] Chiang Y.P., Shimada Y.J., Ginns J., Weiner S.D., Takayama H. (2018). Septal myectomy for hypertrophic cardiomyopathy: Important surgical knowledge and technical tips in the era of increasing alcohol septal ablation. Gen. Thorac. Cardiovasc. Surg..

[117] Smole T., Žunkovič B., Pičulin M., Kokalj E., Robnik-Šikonja M., Kukar M., Fotiadis D.I., Pezoulas V.C., Tachos N.S., Barlocco F. (2021). A machine learning-based risk stratification model for ventricular tachycardia and heart failure in hypertrophic cardiomyopathy. Comput. Biol. Med..

[118] Savariya U., Aponte M.M.P., Nathan S., Zhao B., Radovancevic R., de Armas I.A.S., Kar B., Gregoric I.D., Buja L.M. (2022). Hypertrophic cardiomyopathy with a complex clinical course leading to heart transplantation. Cardiovasc. Pathol..

[119] Goff Z.D., Calkins H. (2019). Sudden death related cardiomyopathies—Hypertrophic cardiomyopathy. Prog. Cardiovasc. Dis..

[120] Mateo J.J.S., Gimeno J.R. (2018). Alcohol septal ablation in hypertrophic cardiomyopathy. Glob. Cardiol. Sci. Pract..

[121] Kogut J., Popjes E.D. (2020). Hypertrophic cardiomyopathy 2020. Curr. Cardiol. Rep..

[122] Brieler J., Breeden M.A., Tucker J. (2017). Cardiomyopathy: An overview. Am. Fam. Physician.

[123] Vaidya K., Semsarian C., Chan K.H. (2017). Atrial fibrillation in hypertrophic cardiomyopathy. Heart Lung Circ..

[124] Yeung C., Enriquez A., Suarez-Fuster L., Baranchuk A. (2019). Atrial fibrillation in patients with inherited cardiomyopathies. Europace.

[125] Garg L., Gupta M., Sabzwari S.R.A., Agrawal S., Agarwal M., Nazir T., Gordon J., Bozorgnia B., Martinez M.W. (2019). Atrial fibrillation in hypertrophic cardiomyopathy: Prevalence, clinical impact, and management. Heart Fail. Rev..

[126] Falasconi G., Pannone L., Slavich M., Margonato A., Fragasso G., Spoladore R. (2020). Atrial fibrillation in hypertrophic cardiomyopathy: Pathophysiology, diagnosis and management. Am. J. Cardiovasc. Dis..

[127] Patten M., Pecha S., Aydin A. (2018). Atrial fibrillation in hypertrophic cardiomyopathy: Diagnosis and considerations for management. J. Atr. Fibrillation.

[128] Weissler-Snir A., Adler A., Williams L., Gruner C., Rakowski H. (2017). Prevention of sudden death in hypertrophic cardiomyopathy: Bridging the gaps in knowledge. Eur. Heart J..

[129] Hindieh W., Adler A., Weissler-Snir A., Fourey D., Harris S., Rakowski H. (2017). Exercise in patients with hypertrophic cardiomyopathy: A review of current evidence, national guideline recommendations and a proposal for a new direction to fitness. J. Sci. Med. Sport..

[130] Marrakchi S., Kammoun I., Bennour E., Laroussi L., Kachboura S. (2020). Risk stratification in hypertrophic cardiomyopathy. Herz.

[131] Jordà P., García-Álvarez A. (2018). Hypertrophic cardiomyopathy: Sudden cardiac death risk stratification in adults. Glob. Cardiol. Sci. Pract..

[132] Lee J.M., Park H.B., Song J.E., Kim I.C., Song J.H., Kim H., Oh J., Youn J.C., Hong G.R., Kang S.M. (2022). The impact of cardiopulmonary exercise-derived scoring on prediction of cardio-cerebral outcome in hypertrophic cardiomyopathy. PLoS ONE.

[133] Ji Q., Wang Y.L., Liu F.Y., Yang Y., Xia L.M., Ding W.J., Lai H., Wang C. (2022). Hypertrophic cardiomyopathy with latent obstruction: Clinical characteristics and surgical results. J. Cardiol..

[134] Butters A., Lakdawala N.K., Ingles J. (2021). Sex differences in hypertrophic cardiomyopathy: Interaction with genetics and environment. Curr. Heart Fail. Rep..

[135] Norrish G., Field E., Kaski J.P. (2021). Childhood hypertrophic cardiomyopathy: A disease of the cardiac sarcomere. Front. Pediatr..

[136] Kitaoka H., Kubo T., Doi Y.L. (2020). Hypertrophic cardiomyopathy ― A heterogeneous and lifelong disease in the real world. Circ. J..

[137] Popa-Fotea N.M., Micheu M.M., Bataila V., Scafa-Udriste A., Dorobantu L., Scarlatescu A.I., Zamfir D., Stoian M., Onciul S., Dorobantu M. (2019). Exploring the continuum of hypertrophic cardiomyopathy—From DNA to clinical expression. Medicina.

[138] Serra W., Vitetta G., Uliana V., Barocelli F., Barili V., Allegri I., Ardissino D., Gualandi F., Percesepe A. (2022). Severe hypertrophic cardiomyopathy in a patient with a homozygous *MYH7* gene variant. Heliyon.

[139] Musumeci B., Tini G., Russo D., Sclafani M., Cava F., Tropea A., Adduci C., Palano F., Francia P., Autore C. (2021). Left ventricular remodeling in hypertrophic cardiomyopathy: An overview of current knowledge. J. Clin. Med..

[140] Marian A.J. (2002). Modifier genes for hypertrophic cardiomyopathy. Curr. Opin. Cardiol..

[141] Orenes-Piñero E., Hernández-Romero D., Jover E., Valdés M., Lip G.Y., Marín F. (2011). Impact of polymorphisms in the renin-angiotensin-aldosterone system on hypertrophic cardiomyopathy. J. Renin Angiotensin Aldosterone Syst..

[142] Pradeep R., Akram A., Proute M.C., Kothur N.R., Georgiou P., Serhiyenia T., Shi W., Kerolos M.E., Mostafa J.A. (2021). Understanding the genetic and molecular basis of familial hypertrophic cardiomyopathy and the current trends in gene therapy for its management. Cureus.

[143] Glavaški M., Stankov K. (2019). Epigenetics in disease etiopathogenesis. Genetika.

[144] Dias K.A., Link M.S., Levine B.D. (2018). Exercise training for patients with hypertrophic cardiomyopathy: JACC review topic of the week. J. Am. Coll. Cardiol..

[145] Nollet E.E., Westenbrink B.D., de Boer R.A., Kuster D.W.D., van der Velden J. (2020). Unraveling the genotype-phenotype relationship in hypertrophic cardiomyopathy: Obesity-related cardiac defects as a major disease modifier. J. Am. Heart Assoc..

[146] Ren J., Wu N.N., Wang S., Sowers J.R., Zhang Y. (2021). Obesity cardiomyopathy: Evidence, mechanisms, and therapeutic implications. Physiol. Rev..

[147] Sun D., Schaff H.V., McKenzie T.J., Nishimura R.A., Geske J.B., Dearani J.A., Ommen S.R. (2022). Safety of bariatric surgery in obese patients with hypertrophic cardiomyopathy. Am. J. Cardiol..

[148] Jansweijer J.A., van Spaendonck-Zwarts K.Y., Tanck M.W.T., van Tintelen J.P., Christiaans I., van der Smagt J., Vermeer A.M.C., Bos J.M., Moss A.J., Swan H. (2019). Heritability in genetic heart disease: The role of genetic background. Open Heart.

[149] Maron B.J., Rowin E.J., Arkun K., Rastegar H., Larson A.M., Maron M.S., Chin M.T. (2020). Adult monozygotic twins with hypertrophic cardiomyopathy and identical disease expression and clinical course. Am. J. Cardiol..

[150] Cashman T.J., Josowitz R., Johnson B.V., Gelb B.D., Costa K.D. (2016). Human engineered cardiac tissues created using induced pluripotent stem cells reveal functional characteristics of BRAF-mediated hypertrophic cardiomyopathy. PLoS ONE.

[151] Mosqueira D., Mannhardt I., Bhagwan J.R., Lis-Slimak K., Katili P., Scott E., Hassan M., Prondzynski M., Harmer S.C., Tinker A. (2018). CRISPR/Cas9 editing in human pluripotent stem cell-cardiomyocytes highlights arrhythmias, hypocontractility, and energy depletion as potential therapeutic targets for hypertrophic cardiomyopathy. Eur. Heart J..

[152] Yadav S., Yuan C.C., Kazmierczak K., Liang J., Huang W., Takeuchi L.M., Kanashiro-Takeuchi R.M., Szczesna-Cordary D. (2019). Therapeutic potential of AAV9-S15D-RLC gene delivery in humanized MYL2 mouse model of HCM. J. Mol. Med..

[153] Nguyen Q., Lim K.R.Q., Yokota T. (2020). Genome editing for the understanding and treatment of inherited cardiomyopathies. Int. J. Mol. Sci..

